# Recent Advances in Mulberry Processing and Drying Technologies: A Comprehensive Review

**DOI:** 10.3390/foods15173165

**Published:** 2026-09-07

**Authors:** Xinge Quan, Qingqing Jiao, Yao Lu, Mochen Liu, Jing Wang, Yudao Li, Shengxiang Zhu, Fuyang Tian, Zhanhua Song, Yinfa Yan

**Affiliations:** 1 College of Mechanical and Electronic Engineering, Shandong Agricultural University, Tai’an 271018, China; 2 Shandong Key Laboratory of Intelligent Production Technology and Equipment for Facility Horticulture, Tai’an 271018, China; 3 Changyi Forestry Development Center, Weifang 261300, China; 4 Shandong Engineering Research Center of Agricultural Equipment Intelligentization, Tai’an 271018, China

**Keywords:** *Morus*, freeze-drying, microwave-vacuum drying, quality retention, process optimisation

## Abstract

Mulberry (*Morus* spp.) leaves, fruits, branches, and root bark are rich in bioactive compounds, including 1-deoxynojirimycin (1-DNJ) and γ-aminobutyric acid (GABA), supporting their potential use in food, medicinal, and feed applications. Their high moisture content, however, makes fresh materials highly susceptible to postharvest quality deterioration, making drying essential for stabilization and high-value utilization. Drying technologies involve trade-offs among efficiency, energy consumption, sensory quality, rehydration, and bioactive-compound retention. This review provides a comprehensive overview of pretreatment and drying technologies for mulberry materials, with particular attention to differences in raw-material characteristics, processing conditions, analytical methods, and reporting bases that limit direct comparisons among studies. Current evidence suggests that low-temperature, low-oxygen, or short-duration technologies, including vacuum freeze-drying, microwave drying, and microwave-vacuum drying, may better preserve quality in certain thermosensitive products, although their benefits remain product- and process-dependent. Hot-air, solar, infrared, heat-pump, and hybrid drying remain practical options for bulk products but require optimization to balance quality, energy efficiency, and scalability. For juice and functional powders, carrier selection, powder properties, and bioaccessibility require further study. Overall, the available evidence is heterogeneous, and some conclusions rely on limited mulberry-specific data or extrapolation from related plant matrices. Future research should emphasize standardized quality evaluation, harmonized reporting, kinetic modeling, multi-objective optimization, online monitoring, energy and carbon-footprint assessment, and industrial-scale validation.

## 1. Introduction

**Figure 1:**
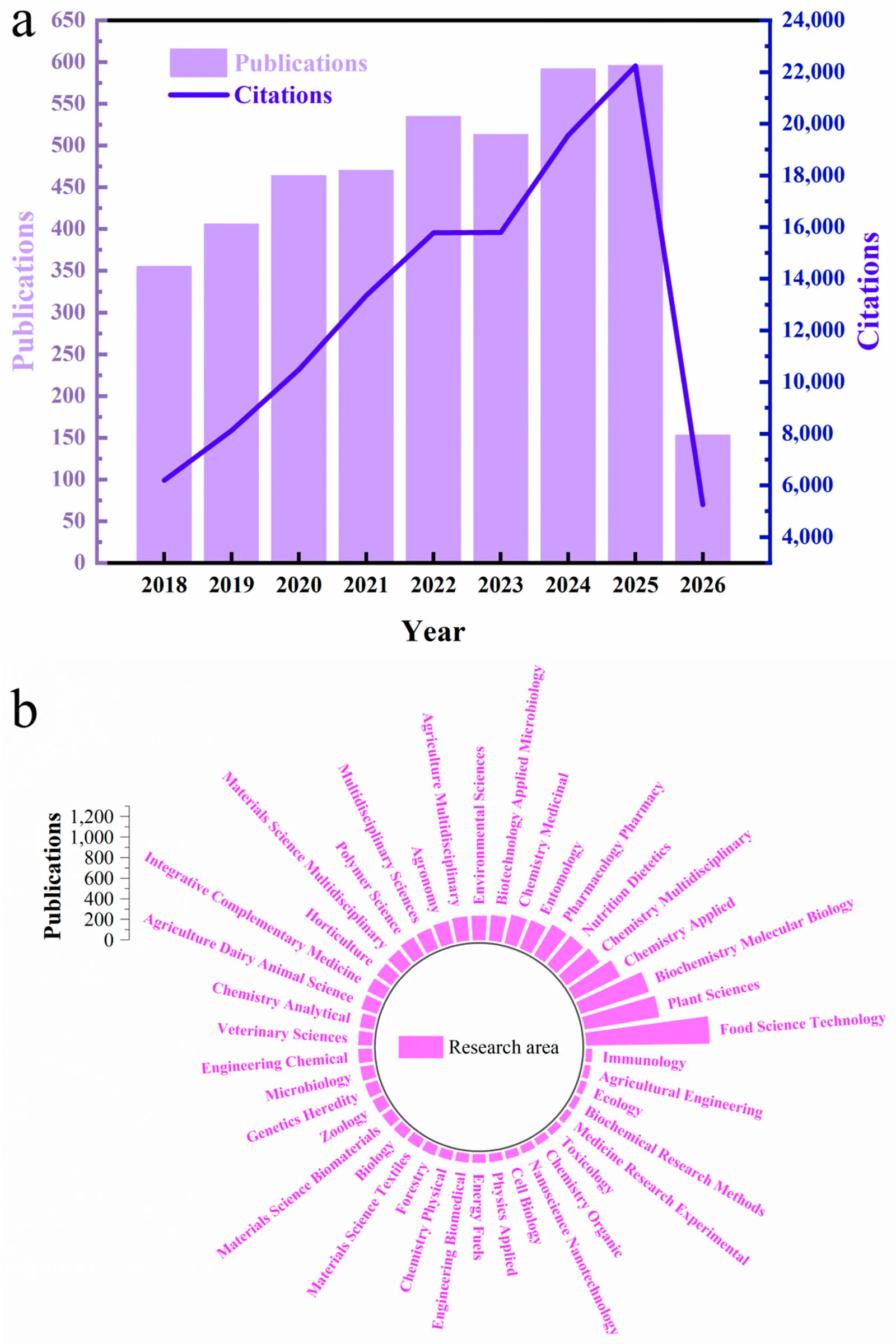
The annual publication and citation trends of mulberry-related studies from 2018 to 2026 (**a**) and the distribution of these publications across major research areas (**b**).

Mulberry trees and their by-products represent versatile biological resources with applications in food, medicine, and animal feed. Traditionally, the sericulture industry has relied primarily on Mulberry leaves (MLs) as feed for silkworms. However, advances in food science, nutrition, and pharmacology have progressively broadened the utilization of MLs, Mulberry fruit (MFs), branches, and root bark for the development of functional foods, natural medicines, feed supplements, fermented products, and cosmetic ingredients [1,2]. Based on data retrieved from the Web of Science Core Collection, mulberry-related research showed an overall increase in both publication output and citation frequency from 2018 to 2025 (Figure 1a), indicating growing research interest in this field. The lower number of publications and citations recorded for 2026 should be interpreted cautiously because the data for this year were incomplete at the time of retrieval. As shown in Figure 1b, mulberry-related studies span a broad range of research areas, with major contributions from Food Science & Technology, Plant Sciences, Pharmacology & Pharmacy, Nutrition & Dietetics, Biochemistry & Molecular Biology, and related agricultural and biomedical disciplines. These results indicate that mulberry research has developed into a multidisciplinary field involving food processing, plant science, nutrition, and health-related applications. Previous studies have demonstrated that mulberry leaf powder, mulberry leaf extracts, and mulberry-derived fermented products may modulate oxidative stress, glucose and lipid metabolism, gut microbiota, and aging-related indicators [2,3]. In addition, the nutritional value of MLs as a feed resource for insects and ruminants has attracted increasing attention [4]. Flavonoids [5,6], phenolic acids [7], alkaloids [8,9], mulberry leaf polysaccharides (MLPs) [10], and proteins are among the major functional components of MLs. In MFs, anthocyanins, phenolic acids, the sugar–acid profile, and volatile compounds collectively contribute to their flavor characteristics and antioxidant capacity [11,12,13]. Different processing techniques exert varying effects on the quality and bioactive components of mulberry materials. Therefore, a key objective of mulberry processing is not only to reduce moisture and extend shelf life, but also to balance product safety, processing efficiency, sensory quality, and the retention of bioactive compounds and overall utilization value.

Postharvest deterioration of mulberry materials exhibits pronounced material-specific characteristics [14]. MLs are thin and soft and possess cuticular and waxy surface structures. Although these structures help living plants resist water loss, they may also increase resistance to outward moisture diffusion during drying [15,16]. In contrast, MFs are characterized by thin peels, high sugar contents, and fragile tissue structures, making them susceptible to tissue collapse, juice leakage, adhesion, browning, and anthocyanin degradation during drying [17]. When heat input is insufficient, the materials remain in a high-water-activity state for an extended period, thereby increasing the risks of enzymatic browning and microbial growth. Conversely, excessive heat input may cause case hardening, localized overheating, degradation of thermolabile compounds, and aroma loss [18,19]. Bioactive compounds in MLs, including 1-DNJ, GABA, MLPs, and flavonoids, as well as those in MFs, including anthocyanins, polyphenols, and volatile compounds, are sensitive to temperature, oxygen, light, pH, and moisture conditions. Therefore, drying processes must achieve an appropriate balance among drying rate, oxidative exposure, and structural preservation. Importantly, the apparent performance of a drying technology cannot be attributed to the drying method alone, because differences in cultivar, maturity or developmental stage, sample geometry, initial moisture content, pretreatment, and drying end points may substantially influence the observed quality responses. Process variables such as temperature–time history, airflow, relative humidity, microwave power density, and vacuum level further complicate direct comparisons among studies.

Current research on mulberry processing has generally evolved along three major research directions. The first direction focuses on raw materials and bioactive compounds, particularly the extraction, stabilization, and functional evaluation of MLPs, flavonoids, 1-DNJ, GABA, proteins, and bioactive peptides from MLs, as well as anthocyanins and polyphenols from MFs. The second direction emphasizes pretreatment strategies, including osmotic dehydration, blanching, ultrasound, microwave treatment, pulsed electric fields, fermentation, phytohormone induction, and encapsulation. These approaches modulate cellular structure, enzymatic activity, and the release of bioactive compounds, thereby creating more favorable mass-transfer conditions for subsequent drying [20,21,22,23,24,25,26,27,28]. The third direction focuses on drying equipment and process intensification, encompassing sun drying (SUD), shade drying (SHD), hot-air drying (HAD), vacuum freeze-drying (VFD), microwave drying (MD), microwave-vacuum drying (MVD), pulsed-vacuum drying (PVD), infrared drying (IR), spray drying (SPD), foam-mat drying (FMD), and multi-field-coupled drying technologies [29,30,31,32,33,34]. Although these studies have generated substantial data, direct comparisons remain difficult because of differences in raw materials, pretreatments, sample geometry, drying conditions and end points, analytical methods, and reporting bases. Variations in extraction procedures and data expression can affect measured bioactive-compound contents, while changes in antioxidant capacity may also reflect altered extractability, assay responses, or Maillard-derived compounds rather than true retention alone. Therefore, apparent differences among drying technologies should be interpreted cautiously.

Based on the Web of Science Core Collection, this review retrieved and analyzed studies on mulberry and related resources published between 2018 and 2026. The search employed combinations of keywords related to mulberry, pretreatment, drying technologies, and quality retention, and was restricted to English-language publications, yielding 4048 records in the initial search. Following screening of titles and abstracts, with full texts examined when necessary, studies unrelated to mulberry processing, drying technologies, quality changes, or bioactive-compound retention were excluded. As this article is intended as a comprehensive review rather than a formal systematic review, the selected literature was thematically reorganized and synthesized. This review first outlines the characteristics of mulberry materials and the major challenges associated with drying, then discusses the effects of pretreatment on moisture migration and quality attributes. It subsequently summarizes advances in conventional drying, emerging drying technologies, powder production, and combined drying processes, and finally discusses product forms, quality evaluation, process monitoring, and industrial-scale applications.

## 2. Quality Criteria for Dried Mulberry Products

**Figure 2:**
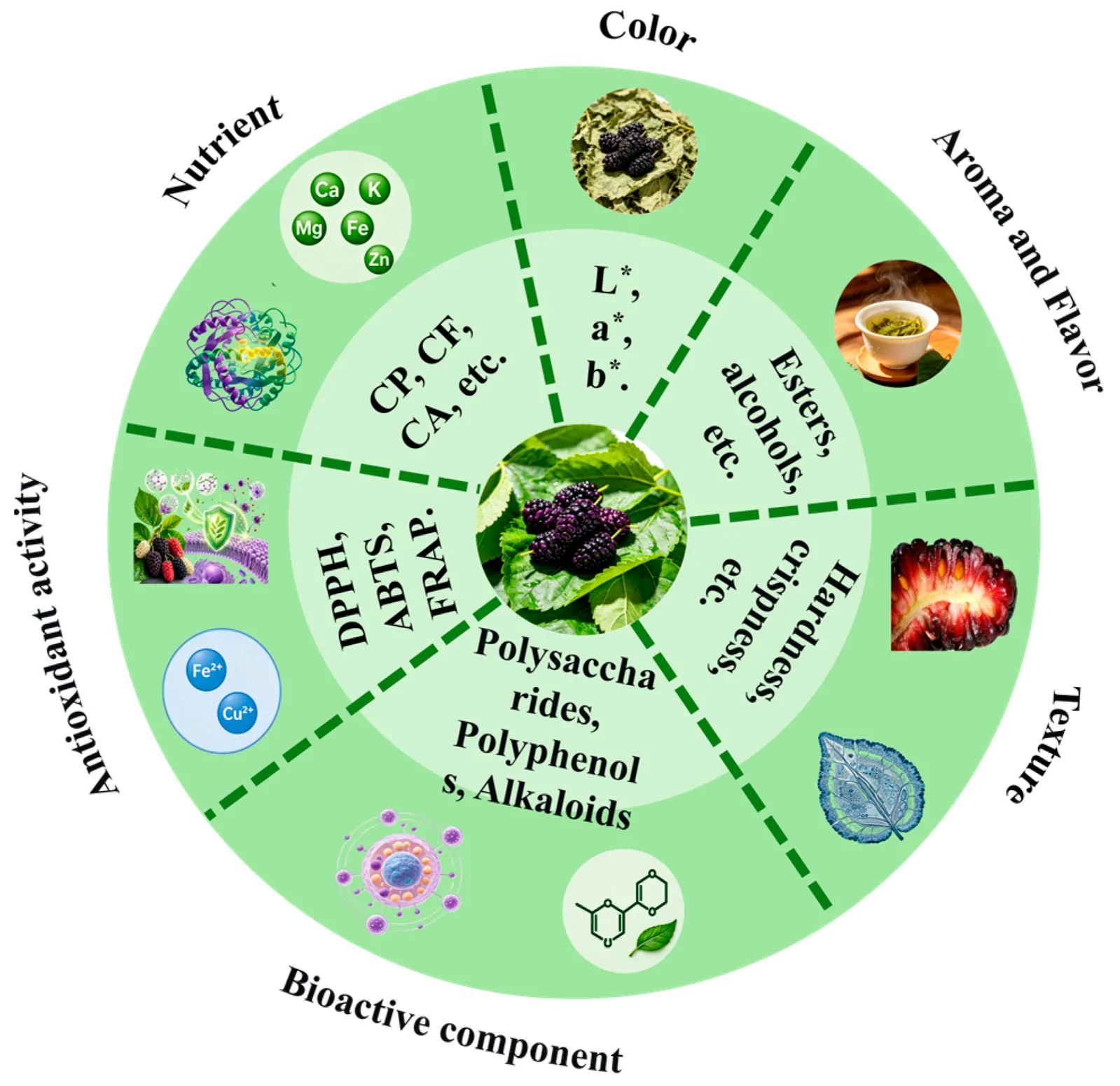
Quality evaluation framework for mulberry-based raw materials and processed products.

Quality evaluation of dried mulberry products should encompass appearance, aroma, texture, nutritional value, bioactive compound retention, safety, and processing suitability, rather than relying solely on moisture content or drying time to assess process performance [35]. For ML tea and ML powder, the retention of a green or yellowish-green color, the stability of infusion color and aroma after brewing, and the preservation of marker bioactive compounds such as 1-DNJ and GABA are important determinants of their functional and commercial value [36]. For dried MFs and MF powders, anthocyanin retention, a purple-red or purple-black appearance, low stickiness, appropriate hardness, good rehydration capacity, and retention of fruity aroma are particularly critical [37,38,39]. For extracts and powder-based ingredients, additional properties should be evaluated, including solubility, hygroscopicity, glass transition temperature, flowability, particle size distribution, encapsulation efficiency, and storage stability [22]. The quality evaluation framework for dried mulberry products is illustrated in Figure 2. In addition, comparisons of drying technologies are mainly valid within single studies or comparable conditions, not as general rankings.

### 2.1. Color, Aroma, and Flavor

#### 2.1.1. Color

Color is an important sensory attribute of mulberry products and mainly reflects changes in pigments and browning reactions rather than overall bioactive-compound retention or nutritional quality [36]. In MLs, color is largely associated with chlorophyll stability, which can be affected by heat, light, acidity, and oxygen exposure [40]. Several studies have reported better green-color retention under microwave pretreatment, VFD, or SPD than under HAD under specific conditions, although the results depend on the raw material and processing parameters [41,42,43,44]. In MFs, color is mainly related to anthocyanin stability, which is sensitive to temperature, pH, light, and oxygen. Low-temperature drying and selected pretreatments may reduce pigment degradation and browning, but color measurements should be interpreted together with direct measurements of anthocyanins and other target compounds when evaluating product quality [37,39,45].

#### 2.1.2. Aroma Profiles and Flavoromics

The aroma profiles of MLs and MFs are composed of hundreds of volatile compounds, primarily including esters, alcohols, aldehydes, and terpenes. Drying not only causes the loss of native low-boiling-point volatile compounds but also generates new thermally derived aroma compounds through lipid oxidation, Maillard reactions, and caramelization. Molecular sensory science approaches, such as solid-phase microextraction coupled with comprehensive two-dimensional gas chromatography–time-of-flight mass spectrometry (SPME–GC×GC–TOF-MS), have enabled detailed characterization of flavor profiles during mulberry drying. Shi et al. [35] identified a total of 232 volatile organic compounds (VOCs) in fresh and VFD-treated MFs. Their results showed that even VFD reduced the concentrations of volatile compounds by at least 21.37%, although it relatively better preserved alcohols and characteristic aldehydic aroma compounds. Yin et al. [38] compared five drying methods and found that high-temperature drying accelerated Maillard reactions and caramelization, leading to the formation of substantial amounts of roasted aroma compounds, such as pyrazines and furans. However, these compounds may mask the original fresh and fruity notes of MFs. These findings indicate that the optimal flavor outcome is not simply determined by maximizing volatile compound retention; rather, the drying route should be selected according to the intended product positioning, such as fresh, green, fruity, or roasted flavor profiles.

### 2.2. Texture and Microstructure

Texture and microstructure determine the chewing experience, rehydration behavior, and subsequent grinding performance of dried mulberry products. The textural properties of MLs and MFs are closely associated with pectin, cellulose, and hemicellulose in the cell wall. Thermal treatment can induce *β*-eliminative degradation of pectin, weaken cell-wall adhesion, and soften tissue structures. Therefore, excessive blanching or high-temperature drying may severely impair product texture, resulting in soft and mushy tissues with poor elasticity after rehydration.

#### 2.2.1. Hardness, Crispness, and Chewiness

Hardness, crispness, chewiness, and springiness are important textural attributes of dried products. Dried MFs and ML tea intended for direct consumption generally require moderate hardness or desirable crispness to facilitate chewing. In contrast, ML powder should be easy to grind. You et al. [39] reported that ultrasound-induced cavitation promoted pectin depolymerization and disrupted cell-wall structures, ultimately reducing the hardness of MFs. MFs dried by MVD exhibited better chewiness than those dried by other methods [41]. In addition, hybrid drying processes incorporating instant pressure-drop technology, such as freeze–explosion puffing drying, can generate uniformly distributed honeycomb-like micropores within MFs, thereby imparting desirable crispness and appropriate hardness [45].

#### 2.2.2. Rehydration Ratio

The rehydration ratio is an important indicator of the ability of dried products to recover a fresh-like state, particularly for dried MFs intended for soup preparation or consumption after rehydration. A high rehydration ratio generally indicates that the drying process causes less damage to the cellular structure and that the product has good water absorption capacity and textural recovery. Studies have shown that VFD-treated products typically exhibit higher rehydration ratios than HAD-treated products because of their porous, sponge-like structures [46]. PVD can also promote the formation of micropores through cyclic pressure variations, thereby improving the rehydration ratio [45].

#### 2.2.3. Surface Microstructure of Mulberry Products

Scanning electron microscopy (SEM) images reveal distinct surface microstructures in MLs subjected to different drying methods. MLs dried by SUD and SHD exhibit relatively smooth surfaces with large cracks, suggesting that these natural drying methods may adversely affect structural integrity and nutrient retention. HAD produces MLs with pronounced structural damage and relatively uniform surface coloration. In contrast, MLs treated by VFD and MD exhibit open stomata and rough, porous surfaces, which may be attributed to low-temperature sublimation and rapid moisture evaporation, respectively. These porous structures may enhance solvent penetration and the extractability of bioactive compounds during subsequent analysis; however, increased extractability should not necessarily be interpreted as greater retention of the native compounds during drying [34]. Similar trends have been observed in MFs. Under HAD, prolonged exposure to high temperatures causes tissue damage and increases hardness. During vacuum drying, moisture is removed under reduced pressure, allowing the samples to retain a honeycomb-like porous structure; however, the external morphology shrinks as a result of water loss. Both HAD and vacuum-dried samples exhibit relatively dense structures, which can be attributed to cellular shrinkage and collapse during drying [45]. In VFD, moisture sublimates directly from preformed ice crystals under low-temperature vacuum conditions, producing a loose and well-organized microstructure. The resulting samples exhibit a fluffy and relatively intact morphology, indicating that low-temperature drying plays a positive role in maintaining porous cellular structures [44].

### 2.3. Major Bioactive Constituents

#### 2.3.1. Polysaccharides

MLPs are among the most abundant bioactive compounds in MLs and exhibit diverse biological activities, including immunomodulatory, hypoglycemic, and antioxidant effects [47]. Owing to differences in drying and extraction technologies, the reported yields of MLPs vary considerably among studies. For example, Zhang et al. [44] reported a polysaccharide yield of approximately 6.12% from shade-dried MLs using hot-water extraction followed by ethanol precipitation, whereas Zhang et al. [48] reported a yield of 6.92% from MLs dried at 60 °C using ultrasound-assisted extraction. Because both the drying and extraction procedures differed between these studies, the reported yields are not directly comparable and should not be interpreted solely as differences in polysaccharide retention during drying. VFD is more conducive to preserving the content and bioactivity of MLPs, whereas prolonged HAD may induce molecular aggregation and broaden the molecular-weight distribution of MLPs, thereby reducing their in vitro antioxidant activity and prebiotic functionality [49].

#### 2.3.2. Polyphenols

Polyphenols mainly comprise two major groups, namely flavonoids and phenolic acids. Flavonoids, including flavonoid glycosides, rutin, quercetin 3-(6-acryloylglucoside), and isoquercitrin, have been identified as major antioxidant compounds in MLs and MFs [29,50]. With respect to phenolic acids, MLs are rich in chlorogenic acid, caffeic acid, and ferulic acid, which exhibit strong antioxidant and anti-inflammatory activities. These polyphenolic compounds are heat-sensitive and readily undergo oxidation and thermal degradation at elevated temperatures. Some mulberry studies have reported lower extractable polyphenol contents after MD or HAD under the conditions investigated [34], although the observed differences may reflect both thermal degradation and changes in compound extractability during processing [51]. In contrast, VFD and IR are more effective in retaining polyphenols, particularly rutin [34,52]. Regarding pretreatment, ultrasound-assisted extraction and ultrasound pretreatment before drying can improve polyphenol retention, including anthocyanin retention, and enhance the antioxidant capacity of dried MFs, owing to cavitation-induced cell disruption and relatively mild thermal effects [53]. Microwave pretreatment also demonstrates good polyphenol-retention capacity, followed by steam blanching and conventional blanching. This advantage may be attributed to the rapid heat transfer and strong penetration capability of microwave and steam-blanching treatments, which enable prompt enzyme inactivation [27].

#### 2.3.3. Alkaloids and GABA

1-DNJ is a distinctive alkaloid present in MLs and exhibits potent α-glucosidase inhibitory activity. It is therefore regarded as an important lead compound for the development of novel hypoglycemic agents and as a functional food ingredient [54,55]. In addition, MLs are rich in glutamic acid and glutamate decarboxylase, and glutamic acid can be converted into GABA under specific stress conditions [56]. GABA exhibits a range of biological activities, including neuroprotective, antihypertensive, antioxidant, anxiolytic, and anti-inflammatory effects. Studies have shown that roller heating, microwave treatment, and steam-blanching pretreatments can result in relatively high 1-DNJ retention in MLs [57,58]. In contrast, soaking MLs in a sodium glutamate solution as a pretreatment provides better GABA retention [26]. With respect to drying-method selection, low-temperature drying is more favorable for retaining 1-DNJ, whereas HAD appears to better preserve GABA [34].

### 2.4. Antioxidant Activity

Antioxidant capacity, as measured by in vitro chemical assays such as DPPH, ABTS, and FRAP, is commonly used to compare the radical-scavenging or reducing properties of mulberry extracts. However, these assays should be regarded as screening tools and should not be interpreted as direct measures of native bioactive-compound retention, in vivo antioxidant effects, or nutritional benefits. In academic studies, in vitro chemical assays, including DPPH, ABTS, and FRAP assays, are commonly used to evaluate antioxidant capacity [34]. Changes in measured antioxidant capacity during drying may be associated with changes in phenolics, flavonoids, and anthocyanins, total flavonoid content, and anthocyanin content. Changes in DPPH, ABTS, or FRAP values may reflect not only the retention or degradation of native compounds but also altered extractability, assay-specific responses, structural transformations, and the formation of Maillard-derived reducing compounds during thermal processing. Under the reported experimental conditions, HAD above 70 °C was associated with lower measured levels of several phenolic compounds and lower in vitro antioxidant capacity of the resulting extracts of the resulting extracts [43,52]. However, thermal effects should be interpreted in relation to the complete temperature–time history rather than nominal drying temperature alone. Drying affects not only small-molecule compounds but also the bioactivity of macromolecular MLPs. A comparative study by Zhang et al. [59] demonstrated that MLPs obtained from VFD-treated MLs exhibited the lowest degree of molecular aggregation and the narrowest molecular-weight distribution. Consequently, the extracted MLPs exhibited higher DPPH radical-scavenging activity than those obtained from conventional HAD under the conditions of that study.

Appropriately applied thermal radiation during drying may not necessarily impair antioxidant activity and may instead promote the release of bound phenolic compounds. Sajeevan et al. [16] reported that, during the processing of Thai ML tea, the combination of far-infrared radiation and hot-air convection (FIR–HA) resulted in higher total phenolic content and overall antioxidant activity than commercially available HAD tea. For certain indices, the values even approached those of fresh MLs, which may partly reflect differences in compound retention and extractability into the cellular matrix and its ability to disrupt bonds associated with macromolecular complexes. With respect to pretreatment, approaches such as ultrasound and microwave treatments that better preserve polyphenols may also enhance the antioxidant activity of the resulting extracts to a certain extent.

### 2.5. Basic Nutritional Components

#### 2.5.1. Proteins and Amino Acids

MLs are rich in proteins and amino acids, with crude protein content exceeding 20% in some cases. They also contain abundant essential amino acids required by humans and animals, making MLs a promising unconventional protein source [60]. Drying temperature has a substantial influence on protein conformation. Moderate thermal treatment can induce partial denaturation of mulberry leaf proteins and expose protease-accessible sites, thereby improving their in vitro gastrointestinal digestibility. However, excessive drying temperatures or prolonged drying durations, as may occur during high-temperature HAD and natural drying methods such as SUD and SHD, can cause extensive thermal denaturation and irreversible protein aggregation. Such conditions may also promote pronounced Maillard reactions between reducing sugars and amino acids, particularly lysine with free amino groups, thereby reducing nutritional value. Studies have shown that mechanical drying methods, including HAD, VFD, and MD, result in higher crude protein retention in MLs, whereas natural drying methods such as SUD and SHD are less effective in preserving crude protein [34]. The relatively short processing time and enclosed treatment environment associated with mechanical drying are more favorable for protein preservation [61]. In contrast, the prolonged processing duration of natural drying often promotes oxidation and protein degradation [62].

#### 2.5.2. Carbohydrates and Soluble Sugars

MFs are rich in soluble reducing sugars, such as fructose and glucose, whereas MLs contain certain amounts of sucrose and starch. During drying, particularly HAD and SPD, the low glass transition temperature (T_g_) of fructose and glucose renders sugar-rich matrices highly susceptible to stickiness and caking when the material temperature exceeds T_g_, thereby severely compromising drying yield. Wang et al. [63] reported that the addition of maltodextrin or whey protein as macromolecular carriers during the SPD of mulberry juice effectively increased the T_g_ of the system and improved the powder microstructure and water solubility index.

#### 2.5.3. Dietary Fiber

MLs and MFs contain substantial amounts of insoluble dietary fiber, including cellulose, hemicellulose, and lignin, as well as soluble dietary fiber, mainly pectin. Dietary fiber constitutes the structural framework of MLs and MFs. Wang, Li, et al. [64] demonstrated that pectin and cellulose in the cell wall undergo natural degradation during MF maturation. Alterations in this macromolecular polysaccharide framework directly result in marked changes in the internal moisture diffusion rate during mid- and short-wave infrared drying. Pretreatment technologies can actively modify dietary fiber structures. For example, ultrasound treatment can induce extensive cleavage of side chains in insoluble pectin within MF cell walls, causing nanoscale depolymerization and conversion into soluble pectin, thereby reducing water binding within the matrix [65]. The dried dietary fiber matrix can also encapsulate bioactive compounds. Yang et al. [2] found that ML powder retaining the native cell-wall dietary fiber barrier exhibited sustained intestinal release of polyphenols. Its effects on gut microbiota modulation and attenuation of aging-related changes were superior to those of directly extracted and purified compounds.

#### 2.5.4. Lipids and Fatty Acids

Mulberry materials generally have relatively low crude fat content, typically below 5%. However, their lipid fractions contain a high proportion of unsaturated fatty acids, such as α-linolenic acid and linoleic acid, making them highly sensitive to processing conditions. During SUD and HAD, prolonged exposure to elevated temperatures in the presence of oxygen can readily initiate free-radical chain oxidation of unsaturated fatty acids. Owing to its dielectric heating characteristics, microwave energy can provide rapid and relatively uniform heating [66]. This rapid heating shortens the duration of high-temperature exposure, reduces excessive moisture loss, helps maintain cellular integrity, and limits the leakage of intracellular components, including lipids. Vacuum treatment can effectively reduce oxygen exposure during drying and thereby minimize lipid oxidative degradation [51]. In addition, Zhao et al. [34] observed that the combination of microwave energy and thermal treatment improved lipid retention in MLs.

#### 2.5.5. Mineral Elements

Mulberry materials are rich in macro- and microelements, including Ca, Fe, Zn, Mg, and K, and may therefore contribute to mineral supplementation, bone health, and anemia prevention. Minerals themselves are relatively heat-stable and generally undergo limited degradation during drying; however, different drying methods vary in their ability to retain specific mineral elements. Zhao et al. [34] reported that SUD was more suitable for retaining P and Zn, VFD was more favorable for Cu and Se retention, and MD better preserved K, Mg, Na, and Mn. In contrast, SHD was less effective in preserving mineral elements. Mineral losses during processing mainly occur during pretreatment before drying. When blanching is applied for enzyme inactivation, substantial amounts of water-soluble mineral salts, particularly K and water-soluble Ca, may leach into the blanching water through diffusion and osmotic transfer.

#### 2.5.6. Vitamins

MFs are rich in vitamin C (VC), whereas MLs contain abundant vitamin E (VE) and various B-group vitamins. Zhao et al. [34] reported that SUD was unfavorable for VC retention. MD and VFD showed superior VE retention, whereas the VE content of SHD-treated samples was lower than that of the other groups. As a water-soluble vitamin, VC is highly susceptible to degradation upon exposure to elevated temperatures and intense light [67]. VC degradation depends on the combined intensity and duration of thermal and oxidative exposure rather than nominal drying temperature alone. Thus, although high thermal exposure during HAD or MD may accelerate VC degradation, the shorter processing time of microwave-based drying can partly offset this effect, whereas prolonged light and oxygen exposure during SUD may also contribute to VC loss [68]. In contrast, lower temperatures and reduced light exposure favor VC preservation, resulting in higher VC contents in SHD- and VFD-treated samples. Nevertheless, exposure to atmospheric oxygen may still induce oxidative degradation, which partly explains the superior performance of VFD in preserving VC [69]. VE is a fat-soluble and relatively heat-stable vitamin. However, the prolonged processing duration and exposure to open environments during SUD and SHD may result in lower VE contents [70]. By comparison, mechanically dried samples, including those subjected to HAD, VFD, and MD, benefit from shorter processing times and relatively enclosed conditions, which contribute to the maintenance of higher VE levels. In addition, the relatively high lipid content of MD-treated samples may partly account for their higher VE content [68]. A study on the MVD of mulberry products by Cong et al. [41] further demonstrated that the vacuum environment not only reduced the evaporation temperature of water but also limited oxidative reactions, resulting in substantially higher VC retention in MVD products than in HAD products.

## 3. Mulberry Pretreatment Technologies

Mulberry materials often require physical and chemical pretreatments before processing to improve processing efficiency and enhance their suitability for subsequent processing. Appropriate pretreatment techniques not only help preserve the color, flavor, and bioactive components of mulberry materials, but also improve product quality stability and processing utilization efficiency.

### 3.1. Chemical Pretreatment and Osmotic Dehydration

**Figure 3:**
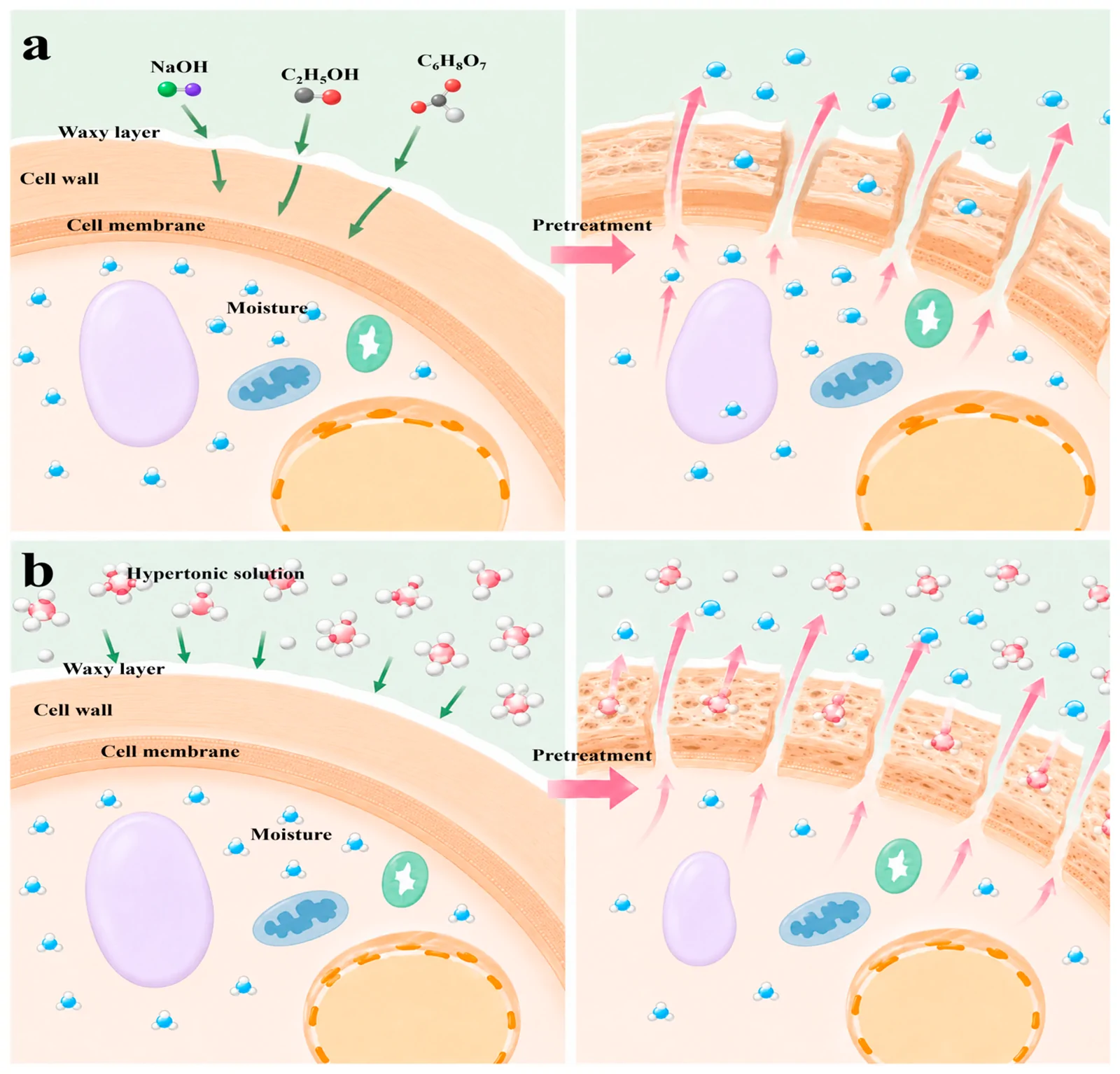
Schematic illustration of water migration in mulberry induced by chemical pretreatment and osmotic dehydration. (**a**) Chemical pretreatment disrupts cellular structures and increases membrane permeability; (**b**) the osmotic pressure gradient drives intracellular water outward.

In fruit and vegetable processing, research on chemical pretreatment has focused primarily on the use of hypertonic solutions, such as sugar and salt solutions. These solutions promote partial water removal through osmotic pressure gradients, thereby improving drying efficiency [20,23,24]. The underlying mechanism is illustrated in Figure 3.

Chemical pretreatment mainly acts by disrupting surface barriers, regulating mass transfer, and inducing stress responses. For example, surfactants such as ethyl oleate can disrupt the microstructure of the cuticular wax layer on fruit and vegetable surfaces and weaken intercellular adhesion through pectin degradation, thereby reducing resistance to moisture migration. In addition, certain chemical agents may act as metabolic substrates or stress-response inducers in the treated materials [71,72]. In MFs, different osmotic solutions, including sucrose, maltose, and sorbitol, exhibit markedly different effects. In the comparative study of Chottamom et al. [73], maltose treatment was associated with relatively higher retention of phenolic compounds and anthocyanins and higher measured antioxidant activity than the other osmotic solutions investigated.

Adabi et al. [74] reported that ethyl oleate pretreatment increased the drying rate of MFs under the conditions investigated. This effect is attributed to the surfactant properties of ethyl oleate, which not only disrupts the microstructure of the surface wax layer but also partially weakens intracellular binding through pectin degradation, thereby increasing the drying rate [75]. Dhiman et al. [76] and Rahimi et al. [77] reported that ultrasound-assisted osmotic dehydration using non-conventional natural sweeteners can remove 30–50% of the initial moisture from MFs. More importantly, the infused solutes can form a dense protective layer on the fruit surface. During subsequent microwave or infrared drying, this barrier effectively buffers anthocyanins against heat-induced stress and substantially improves the texture and shape retention of dried fruits. For MLs, soaking pretreatment with sodium glutamate can significantly promote GABA accumulation. When combined with anaerobic and cold-shock treatments, the GABA content can be increased to approximately three times that of fresh MLs. This improvement is mainly because sodium glutamate serves as a substrate for GABA synthesis and plays a key role in stress-induced GABA accumulation in MLs [50]. Preharvest treatments may also influence the composition of MLs before drying. The application of methyl jasmonate or coronatine to living MLs before harvest can increase total flavonoid and polyphenol contents and alter the nutritional composition matrix of ML powders after drying [28].

During soaking, nutrients in MFs, including polyphenols, pigments, vitamins, and minerals, are prone to migrate into the treatment solution, thereby reducing the nutritional value of the final products. In addition, pretreatment may generate large volumes of wastewater containing chemical agents, soluble nutrients, and pigments, which not only increases the difficulty of recovery and treatment but also imposes a considerable environmental burden [15,71]. Furthermore, residues of alkaline substances, such as sodium hydroxide and sodium carbonate, in the final products may be corrosive and pose potential risks to human health, whereas sulfite residues may irritate the respiratory tract and induce or aggravate respiratory diseases [72].

### 3.2. Blanching and Enzyme-Inactivation Treatments

**Figure 4:**
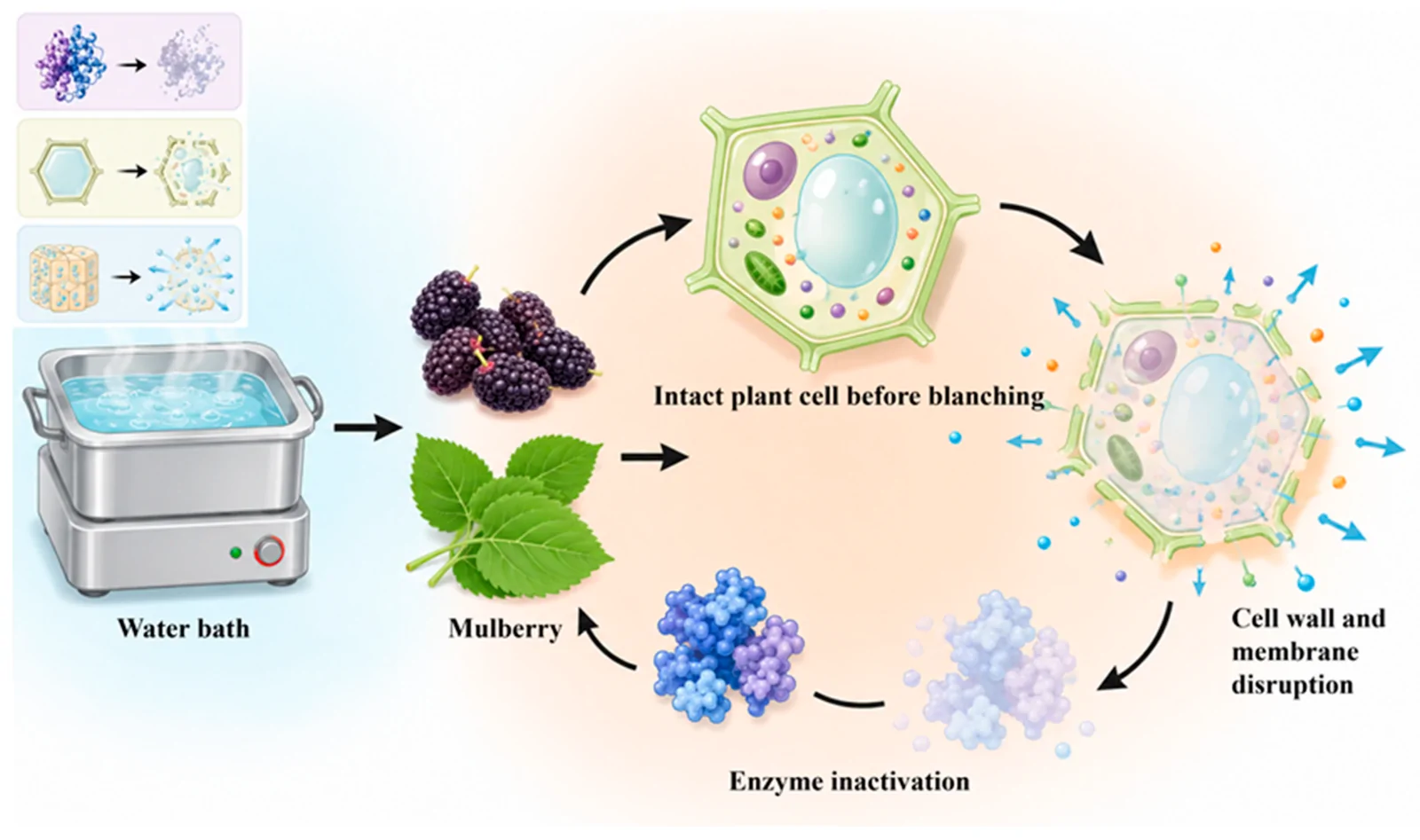
Schematic illustration of the effects of blanching pretreatment on tissue structure and enzymatic activity in mulberry materials.

Blanching and enzyme-inactivation treatments are commonly used as pretreatment methods before drying fruits, vegetables, and traditional Chinese medicinal materials. Their primary purpose is to inactivate enzymes such as PPO and POD through short-duration thermal treatment, thereby limiting enzymatic browning during subsequent processing. However, blanching may also cause thermal degradation or leaching of water-soluble nutrients and bioactive compounds, and its overall effect therefore depends on treatment temperature, duration, medium, and the characteristics of the raw material [78]. The underlying mechanism is illustrated in Figure 4.

Conventional blanching methods mainly include hot-water blanching and steam blanching. Hot-water blanching is operationally simple and relatively low-cost. It can rapidly inactivate PPO and POD, inhibit browning, and soften tissues, thereby facilitating subsequent drying. In contrast, steam blanching reduces direct contact between the material and water, thereby substantially decreasing the loss of water-soluble nutrients. It is therefore considered a milder and more environmentally friendly blanching method. Studies have shown that steam blanching can better preserve total phenolic content and antioxidant activity in MLs [79].

Dhiman et al. [76] applied steam blanching at 80 ± 2 °C for 180 s to improve the quality retention of MFs during subsequent osmotic dehydration and HAD. The results showed that moderate blanching softened tissue structures and increased cell membrane permeability, thereby promoting sugar impregnation and moisture migration and improving the soluble solids content and drying efficiency of the final products. Meanwhile, blanching inhibited enzymatic activity, reduced the oxidative degradation of bioactive compounds such as phenolics and flavonoids, and enhanced the retention of polyphenols and flavonoids as well as antioxidant activity. It was further found that total phenolic content, flavonoid content, DPPH antioxidant activity, and tannin content increased with prolonged blanching time. This may be attributed to the enhanced release of bound bioactive compounds following cellular structure disruption, together with enzyme inactivation. Fixation is a key fundamental step in the processing of ML tea and functional ML products. Its primary purpose is to rapidly inactivate enzymes such as PPO through high-temperature treatment, thereby suppressing enzymatic browning and establishing the basis for desirable appearance quality, including a green infusion and green leaf color. Its processing principle and objective are similar to those of blanching. The fixation methods used in ML tea production and their effects on product quality are summarized in Table 1.

Excessive blanching may cause substantial leaching of water-soluble nutrients, such as MLPs, anthocyanins, vitamin C, and phenolic acids, into hot water, resulting in losses of water-soluble and thermolabile bioactive compounds and nutritional components. It may also promote chlorophyll degradation in MLs, leading to the loss of their fresh green color. In addition, starch-rich materials may undergo starch gelatinization, thereby reducing the subsequent moisture migration rate [83].

### 3.3. Ultrasound Pretreatment

**Figure 5:**
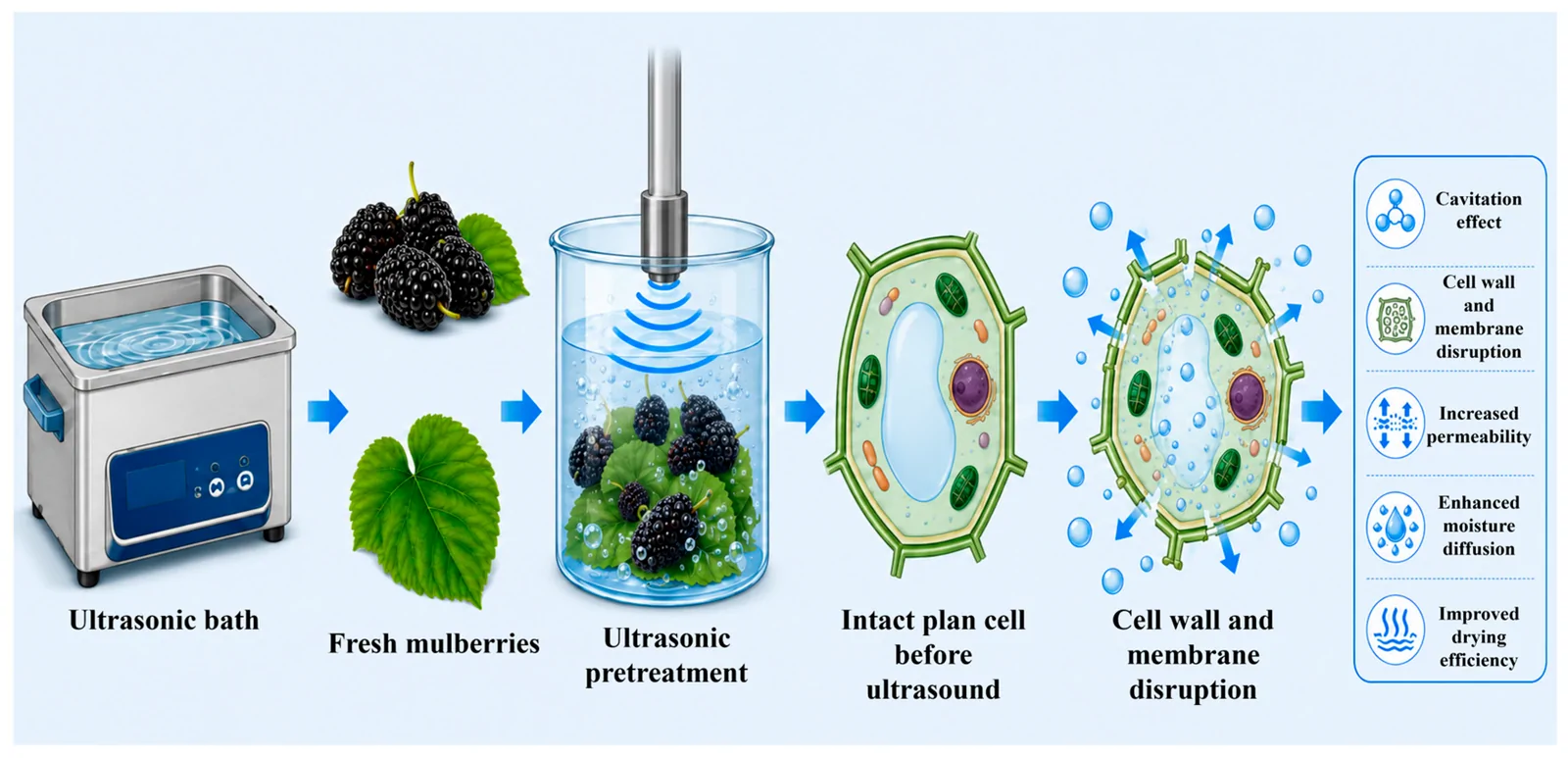
Schematic illustration of the mechanism by which ultrasound pretreatment promotes microchannel formation and moisture migration in mulberry tissues.

During propagation through a medium, ultrasound can generate various physical phenomena, including mechanical vibration, thermal effects, sponge effect, cavitation, and atomization. These effects act synergistically to enhance internal and external heat and mass transfer within materials, thereby effectively improving drying efficiency [84]. Evidence from other fruit and vegetable matrices suggests that ultrasound pretreatment may shorten subsequent drying time and reduce thermal exposure, thereby contributing to quality preservation under appropriate conditions [85]. However, these effects depend strongly on ultrasound parameters and matrix characteristics, and their direct applicability to mulberry materials requires specific validation. The underlying mechanism is illustrated in Figure 5.

Ultrasound-enhanced moisture migration is mainly associated with cavitation and sponge effects. In a liquid medium, ultrasound can induce the formation, growth, and instantaneous collapse of microbubbles, generating localized high temperatures and pressures as well as microjets. These effects exert an etching action on the material surface, disrupt the integrity of the waxy layer, and form microporous channels in the cell wall [86]. Meanwhile, the propagation of ultrasound waves through the solid matrix induces periodic compression and expansion, producing a sponge-like squeezing effect that promotes the outward migration of internal moisture and soluble cellular components. These effects act synergistically to reduce resistance to moisture migration and thereby improve drying efficiency [84,87].

Ultrasound pretreatment has a beneficial effect during the HAD of MLs, effectively increasing the drying rate while better maintaining quality indicators. In MFs, ultrasound pretreatment can modify the nanostructure of cell-wall polysaccharides and improve drying quality [25,77]. Ultrasound-assisted treatments have also been applied to enhance the extraction of mulberry leaf proteins and to investigate changes in 1-DNJ and polyphenol-related metabolism [60]. However, enhanced extractability should be distinguished from actual retention of these compounds during drying. Further studies have indicated that ultrasound pretreatment improves drying efficiency while exerting only limited effects on the color, bioactive compound retention, and antioxidant capacity of final ML products [25,88].

Excessively high ultrasound frequency or prolonged treatment duration may cause nutrient losses during ultrasound pretreatment. These conditions may also lead to severe disruption of cellular structures, resulting in the leaching of pigments and flavor compounds into the treatment medium. Moreover, ultrasound treatment generally requires water as the propagation medium, which raises concerns regarding energy consumption and medium management [85]. Ultrasound-assisted drying also involves relatively complex equipment, including the design and arrangement of ultrasound transducers, equipment size and configuration, and integration with conventional drying systems. Addressing these engineering challenges requires comprehensive consideration of equipment cost, operational convenience, and drying efficiency.

### 3.4. Microwave Pretreatment

**Figure 6:**
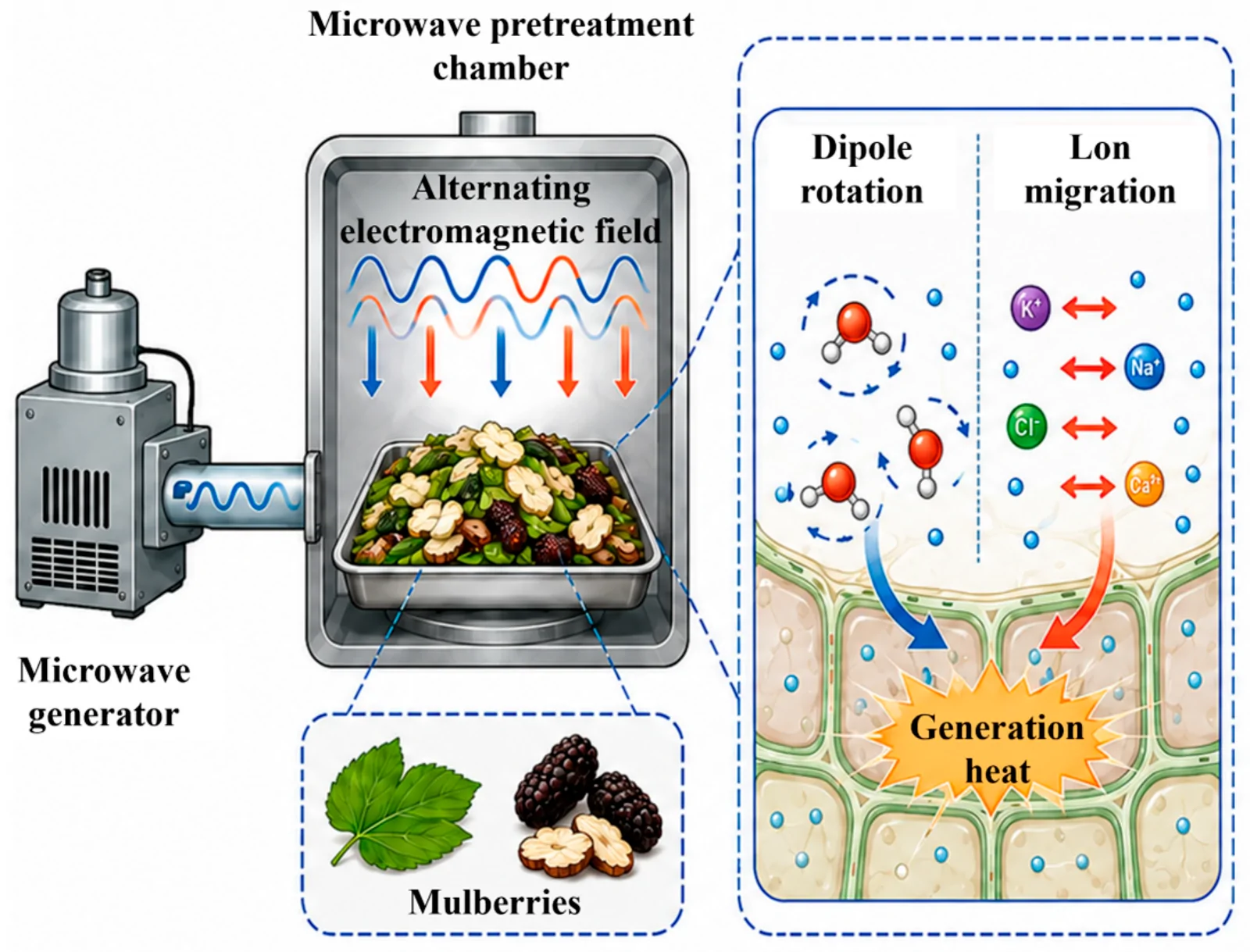
Schematic illustration of microwave-induced internal heating.

In the food industry, microwave energy is widely used as an efficient heat source because it can directly penetrate materials and achieve rapid internal heating through dipolar rotation and ionic conduction [89]. The underlying mechanism is illustrated in Figure 6.

Microwave pretreatment relies on a distinctive selective volumetric heating mode to disrupt the internal physical structure of materials, particularly plant-based matrices. During this process, microwave energy can further act at the molecular level to disassemble or modify target components. These structural and molecular alterations create more favorable kinetic conditions for subsequent separation, extraction, conversion, or drying processes, thereby improving drying efficiency and product quality [90].

Microwave pretreatment and microwave-assisted processing offer the advantages of volumetric heating, rapid temperature rise, and selective interaction with water molecules. Microwave treatment can rapidly reduce the activity of certain enzymes and disrupt cellular structures, potentially increasing the extractability of bound phenolic compounds [89]. Combined microwave–ultrasound pretreatment of mulberry juice increased total phenolic content, total anthocyanin content, and antioxidant capacity under appropriate microwave treatment durations and citric acid addition conditions [91]. This improvement may be attributed to rapid heat generation during microwave treatment, which effectively inactivates PPO and thereby reduces the oxidative loss of phenolic compounds. Positive associations between microwave treatment duration and measured DPPH and ABTS radical-scavenging activities have been reported under certain experimental conditions [89]. However, such increases may reflect altered extractability, assay-specific responses, or the formation of thermally derived reducing compounds rather than greater retention of native antioxidants alone.

Microwave heating often suffers from poor spatial uniformity, and localized overheating or non-uniform treatment can readily occur during large-scale processing, compromising process consistency. In addition, microwave technology requires specialized equipment and involves relatively high initial investment and operating costs. Its effectiveness is also highly dependent on material properties, such as dielectric characteristics and moisture content. Moreover, insufficient control over process parameters, including power, treatment time, and temperature, may lead to the degradation of thermolabile bioactive compounds [89,90].

### 3.5. Pulsed Electric Field Pretreatment

**Figure 7:**
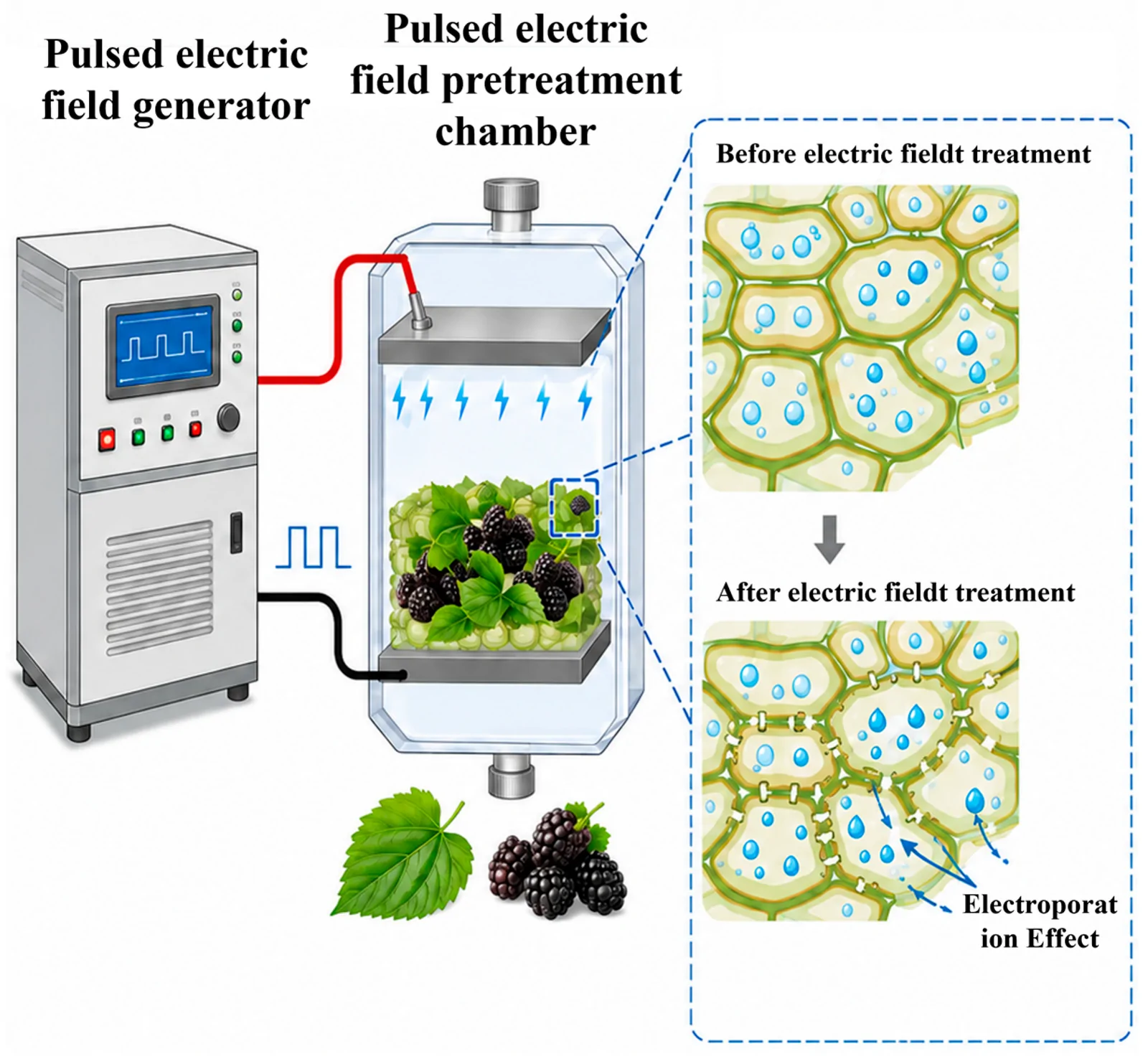
Schematic illustration of cell membrane electroporation induced by pulsed electric field pretreatment.

Pulsed electric field (PEF) pretreatment refers to the application of short-duration high-voltage electrical pulses to food materials and represents an emerging nonthermal processing technology [92]. Although its application in mulberry drying remains limited, PEF shows considerable potential. Its mechanism is illustrated in Figure 7.

PEF relies on short-duration electrical pulses, ranging from nanoseconds to milliseconds, with electric field strengths typically varying from 1 to 10 kV/cm. By applying short high-voltage pulses to materials, irreversible electroporation can be induced in cell membranes, leading to increased membrane permeability, water leakage, and tissue softening [93,94]. Because PEF is performed under low-temperature conditions, it can effectively protect thermosensitive nutrients and bioactive compounds [95,96].

Chaiyana et al. [21] applied PEF as a pretreatment for the extraction and stabilization of bioactive compounds from MLs. This treatment increased phenolic content and enhanced antioxidant, anti-tyrosinase, and anti-hyaluronidase activities, thereby providing a promising upstream processing strategy for the high-value utilization of ML raw materials in cosmetics and cosmeceutical products. In other fruits and vegetables, PEF has been reported to increase drying rates, improve rehydration properties, modulate the structure of freeze-dried tissues, and enhance the nutritional quality of products such as blueberries, thereby providing indirect support for its potential application in the drying of MFs and MLs.

PEF treatment also has several limitations. Although it can effectively inactivate vegetative cells, it is generally less effective against bacterial spores and certain enzymes, such as PPO, which may lead to quality deterioration during storage. In addition, PEF equipment is expensive, and electrochemical reactions caused by electrode corrosion may pose potential safety concerns. Moreover, successful examples of industrial-scale application remain limited. Different processing pretreatment techniques, along with their mechanisms of action and effects on quality, are shown in Table 2.

## 4. Drying Technologies

**Figure 8:**
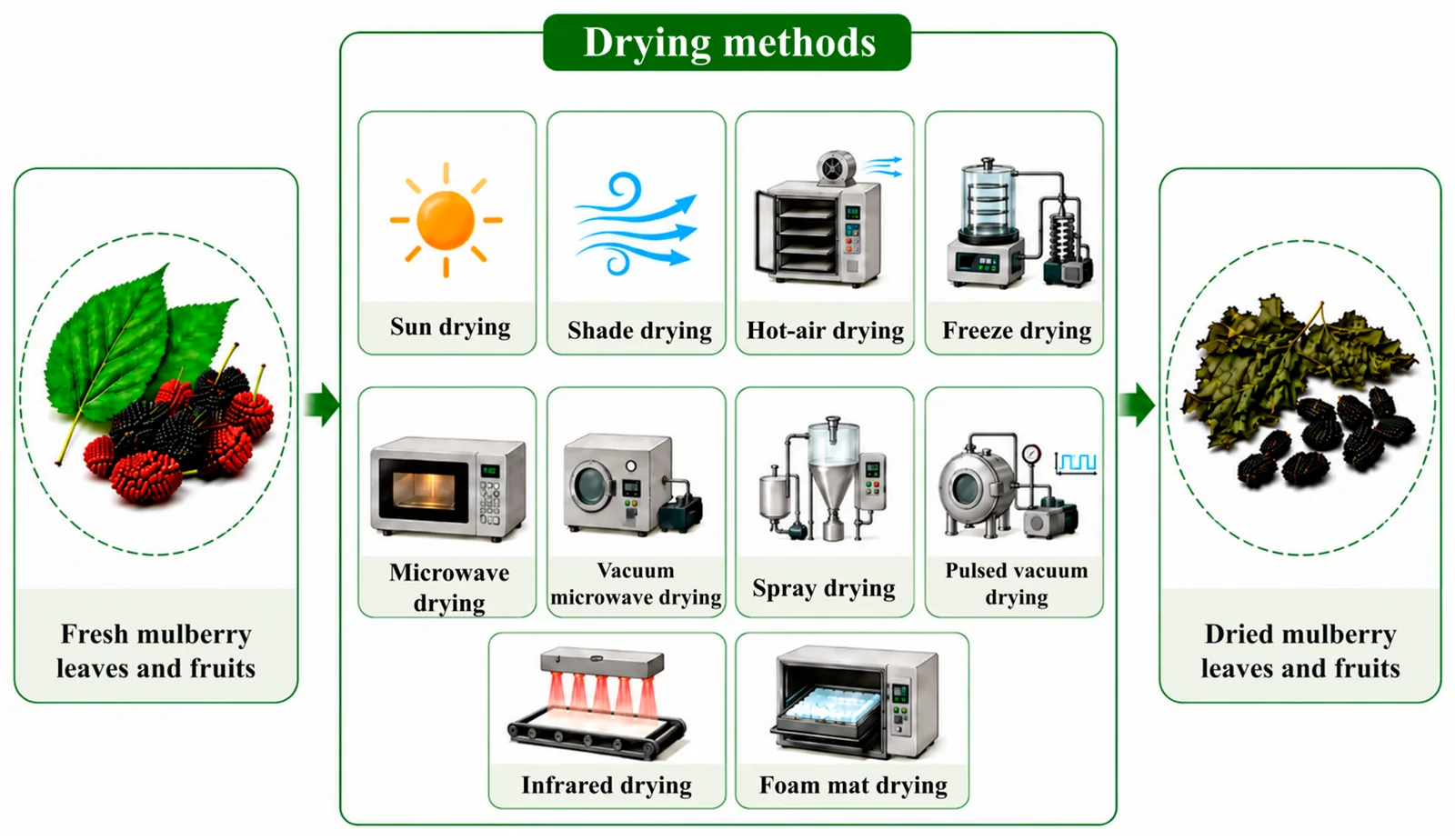
Schematic diagram of the main drying technology routes for MLs and MFs.

**Figure 9:**
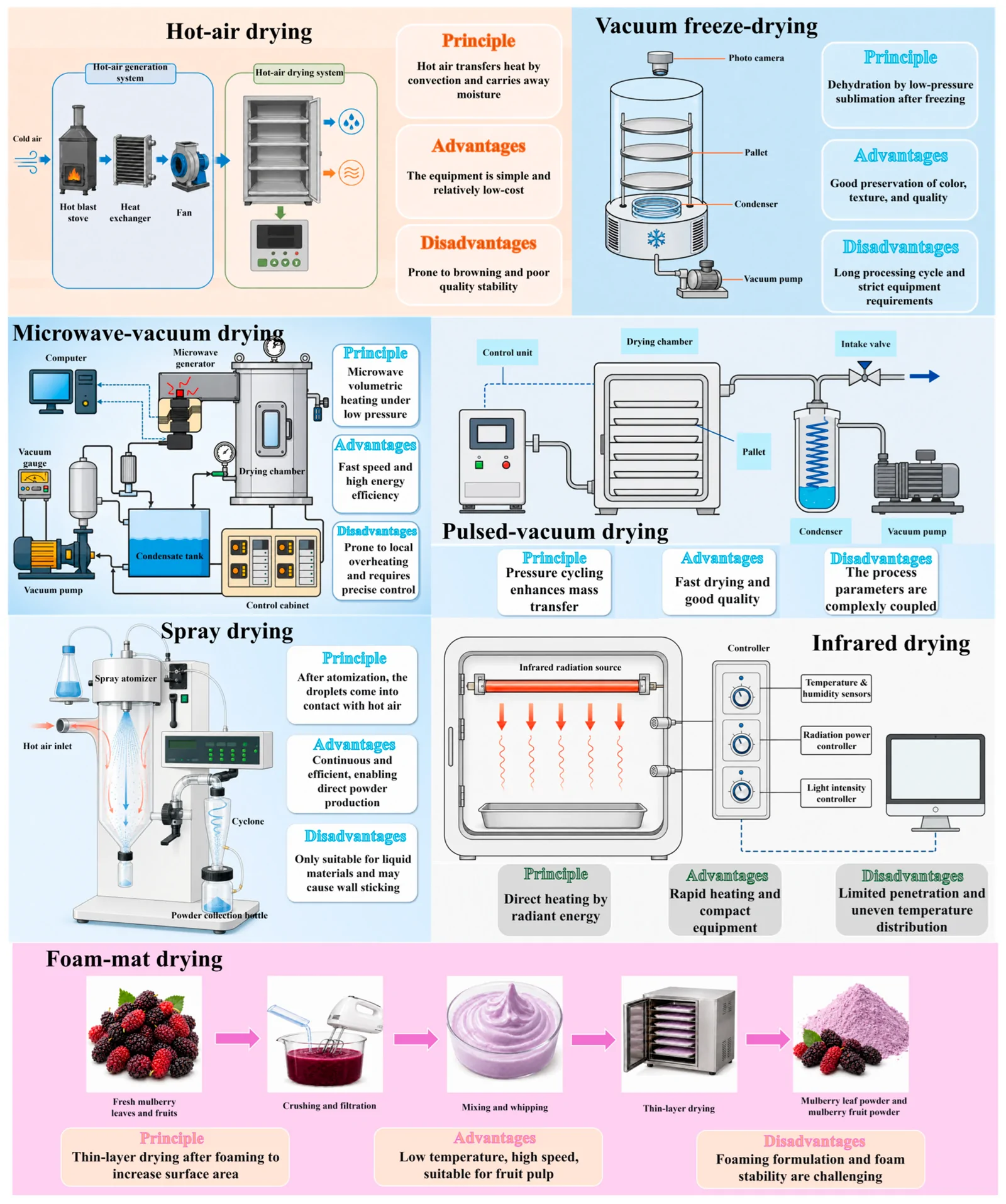
Comparison of the principles, advantages, and limitations of main drying technologies for mulberry materials.

Drying is an important step in the processing and storage of mulberry materials. Different drying techniques have varying effects on the nutritional and bioactive components, color, flavor, and tissue structure of mulberry materials; therefore, selecting an appropriate drying method is essential for improving product quality and utilization value. The main drying technology routes for mulberry products, along with their principles, advantages, and limitations, are shown in Figure 8 and Figure 9.

Drying technologies for mulberry materials can be broadly divided into direct dehydration methods for intact MLs or MFs and product-transformation methods for liquid or semi-liquid matrices. SUD and SHD rely mainly on natural convection and environmental heat and are characterized by low cost but relatively slow and less controllable moisture removal [15,83]. HAD offers greater process controllability, scalability, and compatibility with continuous production through convective heat and mass transfer [98,99]. VFD removes frozen water by sublimation under vacuum and is particularly applicable to thermosensitive products, although its relatively high energy demand and equipment cost remain important limitations [15,100]. MD provides rapid volumetric heating, whereas MVD combines microwave heating with reduced pressure to facilitate moisture removal at lower boiling temperatures; however, their performance strongly depends on microwave power density, vacuum level, sample geometry, loading, and treatment duration [89,101,102]. PVD enhances moisture transport through cyclic pressure changes, while IR supplies radiative energy directly to the material surface and subsurface layers [44,72,103,104]. In contrast, SPD and FMD should be distinguished from direct drying of intact MLs or MFs because they are primarily used to convert juices, extracts, or slurries into powders through atomization or foam-assisted surface-area enhancement, respectively [105,106,107,108]. Overall, no drying technology can be considered universally superior, because product quality depends on raw-material characteristics, sample geometry and loading, temperature–time history, oxygen exposure, airflow or vacuum conditions, microwave power density, and the selected drying endpoint [34,43].

### 4.1. Sun Drying

During SUD, the gradual increase in temperature provides favorable catalytic conditions for endogenous proteases in MLs, promoting the partial degradation of macromolecular proteins into small bioactive peptides and free amino acids [1,57]. This mild dehydration approach can avoid the irreversible loss of essential amino acids, such as lysine, and the formation of advanced glycation end products caused by high-temperature drying, and may enhance the bioactivity of ML proteins in regulating glucose and lipid metabolism [109]. With respect to carbohydrates, the relatively low temperature range of SUD, generally 25–45 °C, can inhibit caramelization, stickiness, and caking, which commonly occur during high-temperature drying of MFs, thereby better maintaining their natural sugar profile. However, prolonged dehydration may consume part of the reducing sugars through continuous respiration, and moisture absorption and mold growth are likely to occur under rainy and humid conditions [64]. From a microstructural perspective, SUD-induced capillary shrinkage can disrupt the spongy tissues of MLs and the cell-wall polysaccharide pore network of MFs, resulting in reduced water-holding capacity, swelling capacity, and rehydration ratio of the dried products [110]. The resulting dense fibrous barrier may also affect the release of functional components, such as polyphenols, during in vitro digestion [2]. In addition, full-spectrum solar radiation, especially ultraviolet radiation, can readily induce photooxidation of unsaturated fatty acids in MLs, generating undesirable flavor compounds such as aldehydes, ketones, and acids [56]. It also accelerates the degradation of chlorophyll into pheophytin, causing SUD-treated ML tea to exhibit a dull, withered, and yellowish sensory appearance [42]. Nevertheless, because SUD does not require liquid-phase pretreatments such as blanching, it can reduce the leaching loss of minerals such as Ca, Fe, Zn, and Mg and enable the relative enrichment of inorganic salts in the ML matrix [45,53]. Studies in animal husbandry have shown that sun-dried MLs, which are rich in Ca, K, and high-quality crude protein, can improve rumen microecology and maintain favorable serological indices in ruminants such as cashmere goats, showing effects comparable to those of ensiled MLs and highlighting their application value in ecological agricultural recycling systems [111].

Prolonged exposure to full-spectrum solar radiation, especially ultraviolet radiation, in the presence of abundant oxygen can induce intense free-radical chain oxidation of membrane lipids in mulberry materials, resulting in the formation of substantial amounts of volatile off-odor compounds. Extremely slow moisture removal intensifies the loss of cellular turgor, leading to irreversible and severe shrinkage of the microstructures of both MFs and MLs. The resulting dense and hardened structural matrix reduces the rehydration ratio of the dried products. Moreover, open-air drying is highly susceptible to physical and biological contamination from dust, insects, birds, and other environmental sources. Under rainy or humid weather conditions, the low drying rate causes the materials to remain within the critical high-water-activity range for an extended period, thereby substantially increasing the risk of mold proliferation and mycotoxin production.

### 4.2. Shade Drying

When comparing multiple drying methods, Yilmaz et al. [68] noted that SHD could most effectively slow the conversion of chlorophyll into pheophytin, enabling MLs to maintain color parameters closest to those of fresh leaves during the early stage of drying, as reflected by lower or more negative a^*^ values. Although light avoidance protects photosensitive pigments, the extremely low dehydration rate of SHD causes materials to remain under high-water-activity conditions for an extended period. Ge et al. [15] reported that, during prolonged dehydration, PPO and POD in MF and ML tissues can remain highly active, promoting the extensive oxidation of phenolic compounds into brown quinone polymers. Consequently, mulberry products subjected to SHD often exhibit a dark-brown appearance in the later drying stage, with significantly lower color uniformity than rapidly dried HAD products. Hu et al. [29] investigated the interactive effects of ML growth stage and drying method and found that although SHD avoids high-temperature damage, the prolonged drying duration allows primary metabolism and respiration to continue after plant tissue detachment. As a result, some antioxidant compounds are consumed as stress-related substrates, and the final total flavonoid retention and DPPH radical-scavenging capacity generally remain intermediate between those obtained by VFD and HAD. Li et al. [43] and Zhang et al. [44] used GC–MS to compare the effects of different drying technologies on volatile compounds in mulberry products and indicated that avoiding high-temperature drying can minimize flavor interference caused by Maillard reactions and caramelization. Yin et al. [38] further confirmed that although SHD-treated mulberry products cannot develop the caramel-like or nutty aromas associated with thermal roasting during HAD, they can retain native low-boiling-point alcohols and esters from mulberry materials, thereby presenting natural grassy and fruity notes.

Owing to its extremely slow dehydration rate, SHD makes MFs and MLs highly susceptible to contamination by fungal spores during drying. Particularly in seasons with high relative humidity, SHD-treated products are prone to deep-seated mold growth and mycotoxin formation [75].

### 4.3. Hot-Air Drying

The drying kinetics of HAD are generally dominated by the falling-rate drying stage. During the later stages of HAD, internal moisture diffusion can become rate-limiting, potentially prolonging thermal exposure depending on air temperature, velocity, sample thickness, loading density, and the selected drying end point [100,112,113]. This imbalance in mass transfer results in severe physical and structural damage. Because surface moisture evaporates much faster than it can be replenished from the interior, MFs undergo rapid shrinkage during the early stage of drying. SEM observations have confirmed that this process causes irreversible cell-wall rupture and severe structural densification, leading to a sharp decrease in the rehydration ratio [46]. Physical collapse occurs not only at the cellular level but also in macromolecular structures. Fourier-transform infrared spectroscopy (FT-IR) and high-performance size-exclusion chromatography analyses by Ma et al. [49] revealed that prolonged HAD induced abnormal cross-linking and aggregation of MLP molecular chains in MLs, resulting in a broadened polysaccharide molecular-weight distribution. These structural changes were associated with lower in vitro antioxidant activity of the extracted MLPs than that observed for VFD-treated samples in the same study. However, such differences may reflect changes in molecular structure and extractability as well as assay-specific responses and should not be interpreted solely as evidence of greater native-compound retention after VFD. At the levels of biochemical composition and flavoromics, HAD causes substantial quality deterioration compared with fresh MLs and MFs. Previous studies have reported greater losses of polyphenols around 70 °C and of 1-DNJ at approximately 125 °C under specific experimental conditions; however, these values should be interpreted as study-specific observations rather than universal thermal thresholds [57,114]. Moreover, the sustained high-temperature and oxygen-rich environment can induce irreversible ring-opening hydrolysis and discoloration of anthocyanins [45]. In addition, with the extensive loss of native low-boiling-point alcohols and ester VOCs, high-temperature-induced Maillard reaction and caramelization products, particularly pyrazines and furans, can mask the natural fresh aroma of mulberry products, leading to profound alterations in the flavor profile [44].

HAD offers practical advantages in terms of operational simplicity, process controllability, scalability, and relatively mature industrial implementation. Its effects on mulberry quality, however, depend strongly on air temperature, relative humidity, airflow velocity, sample thickness and loading density, pretreatment, and the selected drying end point. Excessive thermal exposure or poor control of heat and mass transfer may promote case hardening, shrinkage, degradation of thermolabile compounds, and undesirable flavor changes, whereas appropriately optimized HAD conditions may provide an acceptable balance among drying efficiency, product quality, energy consumption, and processing cost. Therefore, HAD should not be regarded as inherently detrimental. Rather, its suitability should be evaluated according to product requirements and the complete process conditions [75].

### 4.4. Vacuum Freeze-Drying

Based on the phase-transition mechanism of in situ ice sublimation, VFD substantially preserves the physical texture and biochemical quality of fresh mulberry materials. At the microstructural level, VFD can reduce surface-tension-induced shrinkage associated with liquid-water removal and has been reported to produce relatively porous structures with favorable rehydration properties [46]. Chen et al. [45] reported that, owing to the absence of severe thermal stress, ring-opening degradation of anthocyanins in VFD-treated black MFs was inhibited, resulting in the highest retention of total anthocyanin content (TAC) and total phenolics. In addition, from the perspective of polymer physics, Ma et al. [49] confirmed that VFD effectively prevented abnormal aggregation of MLPs during drying and maintained their relatively low molecular weight and highly extended conformation, thereby producing the strongest DPPH radical-scavenging capacity in vitro. Using comprehensive two-dimensional gas chromatography–olfactometry–time-of-flight mass spectrometry (GC×GC–O–TOF-MS), Shi et al. [35] found that although drying inevitably caused volatile losses, VFD retained the characteristic alcohols and natural fruity aldehydes in fresh MFs to a greater extent, while avoiding caramelization-derived off-flavors and Maillard-type roasted notes induced by high-temperature processing. Furthermore, for pharmaceutical and cosmetic applications, Nangare et al. [115] prepared a transfersomal gel loaded with ML extract using freeze-drying technology. The freeze-drying process preserved the anti-tyrosinase activity of the extract while conferring long-term storage stability to the nanovesicles, providing a reference for the application of MLs in anti-aging transdermal delivery systems.

VFD requires the simultaneous operation of high-power vacuum pump systems and deep-cooling cold-trap systems; therefore, its unit energy consumption and equipment depreciation costs are typically 4–6 times higher than those of HAD [116]. In addition, VFD is characterized by low drying efficiency, long drying cycles, and strict requirements for pre-freezing, making it difficult to meet the high-throughput processing demands of bulk agricultural products [75]. For ready-to-eat dried MF snacks, although the highly loose sponge-like texture produced by VFD facilitates chewing, it may lack the crispness preferred by consumers [45].

### 4.5. Microwave Drying and Microwave-Vacuum Drying Technologies

MD is driven by high internal vapor pressure generated within the material, which increases the moisture diffusion coefficient and substantially shortens the drying period [117,118]. At the microscopic level, high-pressure internal vapor can induce a micro-explosion puffing effect when it breaks through cell walls, thereby transforming the dense tissue structure of mulberry materials into a sponge-like microporous network. This physical restructuring not only effectively overcomes the case-hardening defect commonly observed in conventional drying processes but also imparts dried products with a high rehydration ratio and desirable crisp texture [45,119]. In terms of biochemical quality, MVD effectively inhibits oxidative browning through a low-pressure and oxygen-deficient environment and maintains drying-related phase transitions within a low-temperature safety range. This technology enables high retention of antioxidant compounds, such as total phenolics and VC [41]. Moreover, in response to the loss of low-boiling-point aroma compounds and the induction of Maillard reactions caused by high temperatures, Yin et al. [38] and Zhang et al. [44] indicated that MVD, owing to its short heating duration and low-temperature environment, effectively avoids the formation of pungent roasted or burnt odors. It also retains native aldehydes, esters, and other volatile organic compounds to a greater extent, allowing the final dried products to better preserve the original fresh aroma of MLs and MFs.

The main limitations of MD include nonuniform heating, which may generate cold and hot spots; limited penetration depth, which reduces drying efficiency for thick materials; stringent equipment-shielding requirements; and difficulty in continuous production [89]. Although MVD mitigates some thermal-damage problems, it also faces additional challenges, including substantially higher equipment costs, the need to control the risk of glow discharge, foaming and splashing in high-sugar or high-viscosity materials, a predominantly batch-type operation mode, and the existence of an optimal vacuum-pressure window [101,102].

### 4.6. Spray Drying

Mulberry extracts generally exhibit a low glass transition temperature (T_g_), making them prone to wall sticking and caking under high-temperature conditions, which consequently reduces powder recovery. The addition of macromolecular carriers is a key strategy to address this issue. Animal-derived proteins or maltodextrin can improve the physicochemical properties of powders by reducing hygroscopicity and enhancing water solubility. Maltodextrin can also buffer thermal shock and promote the retention of antioxidant bioactive compounds, including anthocyanins [63,120]. To balance drying efficiency with the preservation of thermosensitive compounds, Samborska et al. [121] introduced dehumidified-air-assisted SPD, in which the moisture content of the drying medium was reduced in advance to enhance the mass transfer driving force, thereby allowing lower inlet and outlet air temperatures during the drying of white mulberry molasses. This process not only reduced the oxidative degradation of polyphenols but also increased powder recovery to over 90% with the assistance of prebiotic carriers. Spray microencapsulation has considerable application potential in targeted biological delivery and microbiota modulation. The physical shell layer can protect flavonoids against strongly acidic gastric fluids and enable targeted intestinal release and improved absorption [122]. In terms of process integration, Insang et al. [22] combined ultrasound-assisted extraction with stable encapsulation by SPD, thereby improving the overall quality and shelf life of ML extracts. In addition, given the prebiotic properties of MLPs, Aryaee et al. [123] co-spray-dried MF juice with *Lactiplantibacillus plantarum*. By using added MLPs and exogenous carriers to construct a composite wall system, thermal buffering was provided for lactobacilli, resulting in a synbiotic fruit juice powder with both high viable cell counts and strong antioxidant activity. This strategy provides an engineering reference for the commercial application of whole mulberry resources in gut microbiota intervention.

SPD is associated with high equipment energy consumption and relatively low thermal efficiency, and high-temperature operating conditions may lead to activity losses in thermosensitive core materials [106]. In addition, the controllability of particle morphology and particle size distribution remains limited. Some systems exhibit relatively low encapsulation efficiency, and the resulting powders are prone to wall sticking, moisture absorption, and caking, thus requiring careful matching between formulation design and process parameters [105,124].

### 4.7. Pulsed-Vacuum Drying

Because intense water vaporization removes a large amount of latent heat, the material undergoes pronounced self-cooling during the vacuum phase. This mechanism ensures that the core temperature of MFs and MLs remains within a safe low-temperature range, typically below 55 °C, thereby avoiding the risk of localized overheating associated with constant-temperature HAD [125]. Chen et al. [45] and Zhang et al. [44], when comparing several emerging drying technologies, reported that MFs and MLs subjected to low-temperature and low-oxygen-stress conditions showed limited ring-opening degradation or thermal cleavage of thermosensitive anthocyanins, flavonoids, and VC. For MLs, PVD can effectively maintain the molecular stability of 1-DNJ. For black MFs, PVD-treated fruits not only retain high total phenolic content (TPC) and antioxidant activity but also show no severe browning according to color parameters (L*, a*, and b*).

Under alternating pressure conditions, oxygen may re-enter the micropores of the material during the atmospheric-pressure phase, thereby intensifying oxidative degradation; therefore, costly inert-gas protection may be required. In addition, the intense mechanical stress generated during pressure cycling may cause structural tearing and collapse in fragile fruits. Meanwhile, high-frequency vacuum–atmospheric pressure switching imposes stringent fatigue-resistance requirements on the drying chamber and valves, resulting in persistently high equipment maintenance costs [44,72].

### 4.8. Infrared Drying

Wang et al. [64] investigated the kinetic differences in medium- and short-wave IR drying of black MFs at different maturity stages. Maturity-mediated degradation of pectin and cellulose effectively loosens the macromolecular framework of the cellular matrix and significantly reduces resistance to moisture diffusion, thereby endowing highly mature MFs with higher effective moisture diffusivity and shorter drying periods. Kipcak and Doymaz [117] confirmed that the drying curves of black MFs under IR are also dominated by the falling-rate period and that mathematical models such as the Midilli et al. model can accurately predict their drying kinetics. In terms of quality retention, In some comparative studies, IR-treated samples showed relatively high measured phenolic and flavonoid contents and antioxidant activity, possibly owing to shorter thermal exposure [52]. However, differences in extraction procedures and assay responses should be considered when interpreting these values as evidence of compound retention. However, considering the physical limitations of single IR, particularly its limited penetration depth and tendency to induce surface scorching, IR–HAD offers an effective solution. This multi-field-coupled system not only substantially reduces energy consumption but also balances uniform internal moisture removal with good preservation of fruit appearance and lightness (L*) values, indicating broad applicability in high-quality drying engineering for agricultural products [126,127].

The penetration depth of infrared radiation is limited, usually only a few millimeters, which prevents effective internal heating of thick or stacked materials, such as piled MLs [104,128]. In addition, the linear propagation of infrared radiation can cause pronounced shadowing effects, leading to uneven surface heating of materials [117]. Because the surface layer instantaneously absorbs a large amount of radiant energy, the epidermis of the material is prone to scorching and excessive browning in the absence of external airflow for heat dissipation and moisture removal.

### 4.9. Foam-Mat Drying

MF juice is rich in fructose, which has a very low glass transition temperature (T_g_). HAD or SPD may therefore lead to severe stickiness, agglomeration, and wall sticking. The porous structure formed by FMD not only accelerates moisture removal but also produces a crisp, sponge-like dried sheet that is easy to pulverize, thereby overcoming the stickiness problem associated with high-sugar materials. Khatri et al. [129] systematically optimized the FMD process for black MF juice. Their results showed that an optimized foaming-agent formulation could successfully produce black MF juice powder with excellent shelf stability. In that study, FMD required lower equipment investment and energy input than VFD and showed higher measured color retention and water solubility than the SPD treatment examined. Thuy et al. [32], in optimizing FMD parameters for mulberry extract, noted that the protein and polysaccharide films formed by foaming agents at the gas–liquid interface provided physical protection for encapsulated bioactive compounds, similar to microencapsulation. Combined with relatively low drying temperatures, FMD can efficiently retain polyphenols and antioxidant compounds in mulberry extracts.

Foam stability is highly sensitive to temperature and the initial pH of the material. If foam collapse occurs during the early stage of drying, the drying rate will decrease sharply and may lead to caramelization of the material [130]. In addition, the large amounts of exogenous proteins or polysaccharides added to promote foaming may reduce the purity of mulberry products to some extent and potentially alter their native flavor [131]. The mechanisms of major drying technologies for mulberry products and their effects on quality are presented in Table 3.

## 5. Optimization Strategies for Mulberry Processing Technologies

MLs, MFs, and mulberry by-products hold considerable potential for the development of food, medicinal, feed, and functional ingredient products. However, their processing suitability differs markedly. MLs are edible and medicinal plant materials, and their major processing targets include ML tea, ML powder, ML extracts, functional beverages, feed protein, and functional food ingredients. In contrast, MFs are high-moisture berry materials characterized by high sugar–acid contents and abundant anthocyanins. Their major processing directions include dried MFs, MF juice, MF powder, MF fruit leather, natural pigments, fermented beverages, and antioxidant functional ingredients. Therefore, the optimization of mulberry processing technologies should not follow a single technical route. Instead, tiered process designs should be developed according to raw material type, target product, key bioactive compounds, and industrial-scale production cost. The following processing strategies should therefore be regarded as product-oriented options derived from the currently available evidence rather than universally optimal solutions. Their applicability depends on cultivar, raw-material characteristics, pretreatment, process parameters, drying end points, analytical criteria, production scale, and economic constraints.

### 5.1. Recommendations for Optimizing Mulberry Leaf Drying Processes

Drying strategies for MLs should be selected according to the intended end use. For ML tea, priority should be given to balancing color, aroma, taste, and bioactive compound retention. A processing route involving graded harvesting, rapid enzyme inactivation, low- to moderate-temperature drying, and light-proof, moisture-resistant packaging is recommended. Microwave, steam, or roller heating may be selected for enzyme inactivation depending on the desired flavor profile, whereas HAD, IR-assisted HAD, or low-temperature dehumidification drying may be suitable for the drying stage [42,56,83]. For premium products, metabolomics and aroma analysis can be integrated to optimize flavor quality [26,28,56].

For ML powder intended for food applications, a processing route comprising low-temperature washing, rapid enzyme inactivation, low-temperature HAD or VFD, ultrafine grinding, particle size classification, and moisture-resistant packaging may be adopted. When enrichment of GABA, 1-DNJ, or flavonoids is desired, targeted pretreatments, including monosodium glutamate soaking, anaerobic treatment, cold shock, ultrasound, or fermentation, may be applied before drying to enhance the accumulation of these bioactive compounds [26,56,59,72]. However, increases in the measured contents of these compounds should be interpreted cautiously because they may reflect metabolic accumulation, altered extractability, or both. Direct comparative evidence for different pretreatment–drying combinations remains limited.

For feed applications or protein resource utilization of MLs, process objectives may place greater emphasis on drying efficiency, protein preservation, mold control, and cost reduction. HAD, solar-assisted drying, or HAD–IR hybrid drying can be employed; however, excessively high temperatures should still be avoided to prevent extensive protein denaturation and Maillard reactions [83]. For protein-peptide development, drying should be followed by grinding, protein extraction, and enzymatic hydrolysis. Suitable enzyme systems, such as neutral protease and papain, may be selected, and hydrolysis conditions should be optimized through a comprehensive assessment of the degree of hydrolysis, peptide yield, antioxidant capacity, and anti-inflammatory activity [1]. For this application, critical evaluation should also include protein digestibility and amino-acid availability rather than crude protein content alone. Industrial-scale comparisons of these processing routes remain scarce.

### 5.2. Optimization Recommendations for Mulberry Drying Processes

The core challenges in drying MFs are their high moisture content, high sugar and acid levels, abundant anthocyanins, and tissue structures that are highly prone to collapse. For ordinary dried MF products, a process route combining mass-transfer-enhancing pretreatment with medium- to low-temperature HAD–IR hybrid drying is recommended. For example, short-duration ultrasound-assisted osmotic dehydration or mild blanching may be applied first, followed by HAD–IR hybrid drying, to reduce the drying load, shorten drying time, and improve color and texture [73]. The effectiveness of this route depends on pretreatment intensity, solute concentration, sample size and loading density, airflow, relative humidity, and the complete drying temperature–time history. For MF products positioned as high-anthocyanin and high-antioxidant products, VFD, MVD, or low-temperature infrared-assisted drying may be adopted to reduce oxidation and thermal damage [64,83]. For ready-to-eat crispy MF products, technologies such as freeze–explosion puffing, short-duration puffing after VFD, or vacuum microwave puffing may be explored to address the problems of traditional dried MF products, including sticky and soft texture, poor rehydration capacity, and monotonous mouthfeel [110,134]. For these products, internal pressure development, microwave power, vacuum level, residual moisture, water activity, and structural stability should be regarded as important control points.

The drying endpoint of MFs should be determined by comprehensively considering moisture content, water activity, texture, and storage safety. Using only wet-basis moisture content as the endpoint control criterion is insufficient, because high-sugar MFs may still affect shelf life due to their strong hygroscopicity even when their moisture content is relatively low. It is recommended to establish a combined evaluation system involving water activity, glass transition behavior, moisture sorption isotherms, and packaging barrier properties. For MF products with high anthocyanin content, light-proof, low-oxygen, and moisture-resistant packaging materials should be used, and the storage temperature should be kept as low as possible to slow the decline in color and antioxidant capacity [73].

### 5.3. Optimization Recommendations for Extraction and Preparation of Functional Components from Mulberry Materials

The extraction of bioactive compounds from MLs should be differentiated according to the target compounds. Because of their relatively high polarity, water or aqueous ethanol systems have commonly been used for the extraction of 1-DNJ and related alkaloids, combined with membrane separation, resin adsorption, or pH regulation for enrichment [2,26]. Flavonoids and polyphenols can be extracted using aqueous ethanol, ultrasound-assisted extraction, microwave-assisted extraction, or deep eutectic solvent systems [28,71,72]. MLPs are usually obtained by hot-water extraction, ultrasound-assisted extraction, enzyme-assisted extraction, or microwave-assisted extraction, followed by alcohol precipitation, deproteinization, decolorization, and purification [20,23,24]. The development of proteins and bioactive peptides should follow a technical route integrating protein extraction, enzymatic hydrolysis, membrane separation, and activity screening [1].

Existing studies show that response surface methodology, Box–Behnken design, and central composite design have been widely applied to optimize the preparation of ML-derived MLPs, anthocyanins, and extracts. For polysaccharides, bioactivity depends not only on yield but also on molecular weight, monosaccharide composition, uronic acid content, degree of branching, and conformation. Excessively intense ultrasound, microwave, or acid–alkali conditions may alter their structure and thereby affect functional evaluation [20,23,24]. Therefore, MLP extraction should achieve a balance between high yield and the preservation of structure-related activity. For anthocyanin and polyphenol extraction from MFs, pH, temperature, light exposure, oxygen, and metal ions should be carefully controlled. Acidified ethanol systems are generally favorable for anthocyanin extraction and stability, but solvent residues and regulatory requirements must be considered in food industrial applications. For products intended for use as food ingredients or beverages, food-grade solvents, low-temperature short-duration extraction, and light-protected, low-oxygen operation should be prioritized. After extraction, microencapsulation using *β*-cyclodextrin, maltodextrin, gum arabic, protein–polysaccharide composite wall materials, and related carriers can be adopted to improve the stability of anthocyanins against heat, light, oxygen, and storage conditions [73,76,77]. However, differences in extraction procedures, analytical methods, and reporting bases continue to limit direct comparison among studies. Evidence regarding bioaccessibility, long-term stability, and industrial-scale performance also remains limited.

## 6. Challenges and Future Trends

### 6.1. Challenges

First, MLs and MFs are strongly influenced by cultivar, harvest period, maturity, production area, and storage conditions. These factors lead to substantial raw material variability and limited comparability among different studies. Second, most existing process optimization studies use drying time, yield, or the retention of a single bioactive compound as evaluation indicators, whereas a comprehensive evaluation system integrating color, flavor, texture, bioactive compounds, energy consumption, and cost has not yet been established. In addition, the degradation, release, and transformation mechanisms of components such as 1-DNJ, flavonoids, MLPs, proteins, bioactive peptides, and anthocyanins during fixation, drying, extraction, spray-dried powder production, and storage remain unclear, which limits the scientific basis for product functionality. Moreover, although emerging technologies such as microwave treatment, IR, PVD, ultrasound, and PEF have shown potential for improving efficiency and quality, most studies are still at the laboratory stage. Further breakthroughs are needed in industrial scale-up, continuous control, equipment cost reduction, and standardized application. Future studies should strengthen raw material grading, online process monitoring, multi-objective optimization, and real shelf-life evaluation, thereby promoting the transformation of mulberry processing from experience-based operation toward precise, intelligent, green, and low-carbon manufacturing.

### 6.2. Future Trends

The future development of mulberry processing and drying technologies should shift from simple drying and preservation toward quality-oriented regulation, precise retention of bioactive compounds, and green intelligent manufacturing. Considering the high moisture content of MLs and MFs, their abundance of thermosensitive bioactive compounds, and their distinct tissue structures, future research should place greater emphasis on the combined application of pretreatments such as ultrasound, PEF, osmotic dehydration, short-duration microwave treatment, and fixation/blanching with drying technologies such as HAD, IR, vacuum drying, MD, VFD, and PVD. Such hybrid approaches can enhance moisture migration, shorten drying time, and reduce energy consumption. At the same time, a multi-indicator evaluation system should be established based on the stability of alkaloids, flavonoids, MLPs, proteins, and bioactive peptides in MLs, as well as anthocyanins, polyphenols, and volatile flavor compounds in MFs. This system should integrate color, aroma, texture, bioactive compound retention, antioxidant capacity, water activity, and storage stability. With increasing demand for products such as ML tea, ML functional powder, VFD-treated MFs, MF juice powder, natural pigments, and functional ingredients, future processing equipment should also have greater flexibility and continuous-processing capacity. In addition, online monitoring and intelligent regulation of processing should be achieved by integrating near-infrared spectroscopy, hyperspectral imaging, machine vision, low-field nuclear magnetic resonance, and machine learning.

## 7. Conclusions

In summary, mulberry processing should be tailored to the characteristics of MLs and MFs, their target products, and key quality attributes rather than following a single processing route. For MLs, attention should focus on chlorophyll, 1-DNJ, GABA, MLPs, flavonoids, proteins, and flavor quality, whereas MF processing should emphasize anthocyanins, polyphenols, texture, rehydration, volatile compounds, and microbial stability.

The available evidence does not support a universal ranking of drying technologies. HAD, SUD, and IR may provide practical options for relatively low-cost or large-scale processing, whereas VFD, MVD, and PVD have shown favorable quality retention for certain thermosensitive products. However, these advantages depend on raw-material characteristics, pretreatment, temperature–time history, drying end point, equipment conditions, and analytical methods. Differences in extraction procedures and reporting bases also limit direct comparison of bioactive-compound retention, while antioxidant assays should not be interpreted as direct measures of native compound retention. Therefore, evidence from direct mulberry studies should be distinguished from findings extrapolated from other plant matrices or mechanisms requiring further validation.

Future research should emphasize standardized quality evaluation, harmonized reporting, multi-objective process optimization, online monitoring, kinetic modeling, and industrial-scale validation. Techno-economic and life-cycle assessments are also needed to balance product quality, energy consumption, production cost, and carbon footprint, thereby supporting standardized, intelligent, and sustainable utilization of mulberry resources.

## Figures and Tables

**Table 1:** Effects of different ML fixation methods on sensory quality, bioactive compounds, and antioxidant capacity.

Raw Material	Scale	Fixation Method	Key Conditions	Sensory Quality	Bioactive Compounds	Antioxidant Capacity
MLs	Laboratory	Microwave fixation	Microwave power: 300 W; treatment time: 2.5 min	Bright green leaves, but with residual grassy odor and a relatively simple aroma [80].	High levels of soluble proteins, free amino acids, titratable acids, and chlorogenic acid, with good retention of GABA and some polyphenols [81].	The strongest DPPH radical-scavenging capacity [81].
MLs	Laboratory	Blanching fixation	Blanching temperature: 95 °C; blanching time: 4 min	Glossy leaf surface, markedly reduced grassy odor, and a more pronounced aroma [80].	Increased crude polysaccharides, total alkaloids, and functional monosaccharides, with partial retention of flavonoids and 1-DNJ [82].	Moderate antioxidant capacity.
MLs	Laboratory	Steam fixation	Blanching temperature: 100 °C; blanching time: 2 min	Yellowish tea infusion and infused leaves, enhanced sweetness [80].	High levels of soluble sugars, resveratrol, flavonoids, and polyphenols, with increased free 1-DNJ [81].	Strong ABTS radical-scavenging and metal-chelating capacities [81].

**Table 2:** Mechanisms and quality effects of pretreatment technologies used in mulberry processing.

Raw Material	Scale	Pretreatment Method	Key Conditions	Drying End Point	Evidence Strength	Mechanism	Quality Effects	References
ML tea	Laboratory	Fermentation	8 g mulberry tea + 100 g sugar in 1 L water at 90 °C for 5 min; 10% kombucha starter; 25–28 °C; 14 d	MC: 7% d.b.; aw: NR	Direct–limited	Promotes the release of bound phenolics and regulates the formation of GABA, 1-DNJ, and aroma compounds.	Increases 1-DNJ, GABA, phenolic, and flavonoid contents, but excessive fermentation may cause acidification and darkening.	[26]
White MFs	Laboratory	Ethyl oleate + K_2_CO_3_ dipping	soak for 1 min	MC: 17% w.b.; aw: NR	Direct–limited	Weakens the wax layer and increases epidermal permeability.	Shortens drying time and preserves appearance, but the effects of alkaline conditions on anthocyanins should be controlled.	[97]
MFs	Laboratory	Ethyl oleate + citric acid + ascorbic acid	soak for 1 min	MC: 17% w.b.; aw: NR	Direct–limited	Modifies the wax layer structure and promotes moisture migration.	Accelerates drying and improves drying efficiency.	[97]
Black MFs	Laboratory	Ethyl oleate + potassium salts	soak for 1 min	MC: 10% w.b.; aw: NR	Direct–limited	Disrupts the wax barrier and promotes moisture diffusion.	Reduces energy consumption and is suitable for combination with IR, HAD, or vacuum drying.	[74]
MFs	Laboratory	Osmotic dehydration using sucrose, sorbitol, or maltose	Fruit:solution = 1:6; soaking for 6 h	MC: NR; aw: NR	Direct–limited	Uses a hypertonic environment to promote water outflow.	Reduces initial moisture content; maltose is more favorable for maintaining structure and anthocyanins.	[73]
MLs	Laboratory	Blanching	100 °C, 1 min	MC: 6% w.b.; aw: NR	Direct–limited	Inactivates PPO and POD and regulates the conversion of aroma precursors.	Improves color, aroma, and taste, but excessive treatment may cause losses of bioactive compounds.	[27,81]
Black MFs	Laboratory	Blanching	80 °C, 10 s	MC: 10% w.b.; aw: NR	Direct–limited	Softens the peel and cell walls and inactivates oxidative enzymes.	Promotes moisture migration and reduces energy consumption, but may cause losses of anthocyanins and polyphenols.	[74]
MLs	Laboratory	Power ultrasound	130 W; 5, 10, or 15 min	MC: 10% w.b.; aw: NR	Direct–limited	Forms microchannels and reduces moisture diffusion resistance.	Shortens HAD time, reduces energy consumption, and preserves color and bioactive compounds.	[74]
ML extracts	Laboratory	Ultrasound-assisted extraction	100 W	N/A	Direct–limited	Disrupts cell structures and promotes the release of bioactive compounds.	Increases polysaccharide yield and facilitates bioactive compound enrichment and powder production.	[48]
Black MFs	Laboratory	Ultrasound pretreatment	240 W, 10 min	MC: 10% w.b.; aw: NR	Direct–limited	Reduces internal and external mass-transfer resistance.	Shortens processing time and reduces energy consumption, but excessive treatment may cause softening and juice leakage.	[74]
MFs	Laboratory	Ultrasound pretreatment	NR	MC: NR; aw: NR	Direct–limited	Modifies cell wall structure and increases the proportion of free water.	Accelerates drying, promotes phenolic retention, and enhances antioxidant activity.	[65]
White MFs	Laboratory	Ultrasound-assisted osmotic dehydration	Sucrose 25–60%; ultrasound amplitude 10–90%; 10–30 min	MC: NR; aw: NR	Direct–limited	Ultrasound and osmotic pressure synergistically promote water loss.	Increases water loss, reduces sugar gain, and improves color.	[77]
MF juice and pulp	Laboratory	Microwave–ultrasound pretreatment	Microwave: 2450 MHz, 700 W; ultrasound: 25/40/45 kHz, 150 W, 30 min	MC: NR; aw: NR	Direct–limited	Volumetric heating and cavitation synergistically disrupt cells.	Promotes anthocyanin release and increases functional compound contents.	[91]
Black MFs	Laboratory	Microwave pretreatment	200 W, 10 min	MC: 10% w.b.; aw: NR	Direct–limited	Rapid internal heating induces microcrack formation.	Reduces energy consumption, but excessive treatment may cause overheating or scorching.	[74]
ML extracts	Laboratory	PEF-assisted extraction	10 kV/cm, 5 Hz, 1 μs, 20 min, ~0.6 kWh	N/A	Direct–limited	Electroporation increases membrane permeability.	Promotes bioactive compound release and reduces thermal damage.	[21]
ML protein powder	Laboratory	Enzymatic hydrolysis	0.2 mL enzyme + 0.2 mL casein; 20 g/L; 50 mM Tris-HCl, pH 7; 70 °C, 10 min	N/A	Direct–limited	Hydrolyzes proteins into small peptides and exposes bioactive sites.	Enhances antioxidant and anti-inflammatory activities and is suitable for functional powder production.	[3]

**Table 3:** Mechanisms and quality effects of main drying technologies for mulberry materials.

Raw Material	Scale	Drying Method	Key Conditions	Drying End Point	Evidence Strength	Mechanism	Effects on Quality	References
MLs	Laboratory	SUD	37 ± 2 °C to constant weight	48 h	Direct–limited	Solar radiation heats the material, and natural convection removes moisture.	Low cost but time-consuming; prone to oxidation, contamination, and nutrient losses.	[29,34,132]
MFs	Laboratory	SUD	Sun-drying in June, 09:00–17:00	M_e_	Direct–limited	Solar energy provides heat, and convective airflow removes moisture.	Optimized airflow can improve efficiency, but browning, contamination, and quality fluctuations may occur.	[97,133]
MLs	Laboratory	SHD	25 ± 2 °C, 30% RH	10 d	Direct–limited	Natural ventilation drying under low-temperature and light-protected conditions.	Favors VC and color retention, but requires a long time and may cause mold growth and oxidation.	[29,34,132]
MLs	Laboratory	HAD	65 °C; air velocity 1.2 m/s	12 h	Direct–limited	Hot air supplies heat, and moisture diffuses and evaporates.	Mature and scalable, but high temperatures may cause browning, bioactive compound losses, and shrinkage.	[25,29,34,132]
MFs	Laboratory	HAD	70 °C	M_e_	Direct–limited	Surface moisture evaporates, while internal moisture diffuses outward.	Simple equipment, but case hardening, shrinkage, and losses of anthocyanins and vitamins may occur.	[38,44,46]
MLs	Laboratory	VFD	VFD was carried out at −60 °C.	24 h	Direct–limited	Frozen ice sublimates under vacuum.	Effectively preserves structure and thermolabile compounds, but is costly and time-consuming.	[29,34]
MFs	Laboratory	VFD	Vacuum < 50 Pa; cold trap −50 °C	M_e_	Direct–limited	Ice sublimation forms a porous structure.	Good color, rehydration, and anthocyanin retention, but high energy demand and a loose texture.	[44,46]
MFs	Laboratory	MD	2450 MHz; 90, 180, or 360 W	MC: NR; aw: NR	Direct–limited	Microwave internal heating drives moisture migration.	Rapid drying, but excessive power may cause hot spots, scorching, and anthocyanin degradation.	[38,117]
MLs	Laboratory	MVD	2450 ± 15 MHz; 2 kW; 5 min; 75–85 °C; −0.095 MPa	M_e_	Direct–limited	Microwave heating is combined with a low-temperature, low-oxygen vacuum environment.	Favors the retention of proteins, dietary fiber, and polyphenols, but requires advanced equipment.	[34]
MFs	Laboratory	MVD	Surface temperature 40 °C; 500 W; vacuum 0.08 MPa	23 h	Direct–limited	Microwave energy promotes moisture vaporization under reduced pressure and forms a porous structure.	Faster drying with better rehydration and texture, but industrial scale-up remains challenging.	[41,102]
ML slurry	Laboratory	SPD	60 °C; power supply 11 kW	12 h	Direct–limited	The slurry is atomized and rapidly dried in hot air.	Suitable for producing ML powder and improving powder properties and antioxidant capacity.	[122]
Black MF juice	Laboratory	SPD	Aspirator 100%; airflow 36 m^3^/h; inlet 150 ± 1 °C; outlet 85 ± 1 °C	MC: NR; aw: NR	Direct–limited	Juice is mixed with wall materials and atomized into powder.	Improves solubility and reduces hygroscopicity, but phenolic retention depends on the formulation.	[63,120]
MLs	Laboratory	Combined IR/far-infrared–HAD	FIR intensity 5 kW/m^2^; air 40 °C; velocity 1.5 m/s	60 min	Direct–limited	Infrared heating is combined with hot-air moisture removal.	Produces a color similar to fresh leaves and improves phenolic retention and antioxidant activity, but surface overheating should be avoided.	[52]
MFs	Laboratory	IR/IR-convective drying	60 °C; IR power 1125 W; intensity 4.5 kW/m^2^; air velocity 2.0 m/s	MC: 10% w.b.; aw: NR	Direct–limited	Infrared heating is combined with airflow moisture removal.	High drying efficiency, but scorching and excessive browning may occur.	[64,104,126]
Mulberry extracts	Laboratory	FMD	Extract 1:3 water (*w*/*w*); 6% albumin, 0.3% CMC, 1.5% RDM; foaming 10 min	MC: NR; aw: NR	Direct–limited	Foaming forms a porous thin layer and shortens the moisture diffusion path.	Suitable for low-temperature powder production and polyphenol retention, but depends on formulation and foam stability.	[32]
Black MF juice	Laboratory	FMD	Maltodextrin 2–10 g/100 mL; CMC 1–5 g/100 mL; GMS 1–5 g/100 mL; whipping 5 min	Me	Direct–limited	Suitable for low-temperature powder production and polyphenol retention, but depends on formulation and foam stability.	Good anthocyanin and VC retention with lower cost, but additives may affect flavor.	[129]

## Data Availability

No new data were created or analyzed in this study. Data sharing is not applicable to this article.

## Abbreviations

MLs	Mulberry leaves
MF	Mulberry fruit
1-DNJ	1-deoxynojirimycin
GABA	γ-aminobutyric acid
MLPs	Mulberry leaf polysaccharides
SUD	Sun drying
SHD	Shade drying
HAD	Hot-air drying
VFD	Vacuum freeze-drying
MD	Microwave drying
MVD	Microwave-vacuum drying
SPD	Spray drying
PVD	Pulsed-vacuum drying
IR	Infrared drying
FMD	Foam-mat drying
